# Can General Practitioners manage mental disorders in primary care? A partially randomised, pragmatic, cluster trial

**DOI:** 10.1371/journal.pone.0224724

**Published:** 2019-11-07

**Authors:** Sabrina Gabrielle Anjara, Chiara Bonetto, Poushali Ganguli, Diana Setiyawati, Yodi Mahendradhata, Bambang Hastha Yoga, Laksono Trisnantoro, Carol Brayne, Tine Van Bortel

**Affiliations:** 1 Cambridge Institute of Public Health, School of Clinical Medicine, University of Cambridge, Cambridge, United Kingdom; 2 Department of Neurosciences, Biomedicine and Movement Sciences, University of Verona, Verona, Italy; 3 Institute of Psychiatry, Psychology & Neuroscience, King’s College London, London, England, United Kingdom; 4 Centre for Public Mental Health, Faculty of Psychology, Universitas Gadjah Mada, Yogyakarta, Indonesia; 5 Centre for Health Policy and Management, Faculty of Medicine, Public Health and Nursing, Universitas Gadjah Mada, Yogyakarta, Indonesia; IRCCS E. Medea, ITALY

## Abstract

**Background:**

For a decade, experts have suggested integrating mental health care into primary care to help bridge mental health Treatment Gap. General Practitioners (GPs) are the first port-of-call for many patients with mental ill-health. In Indonesia, the WHO mhGAP is being systematically introduced to its network of 10,000 primary care clinics as an add-on mental health training for pairs of GPs and Nurses, since the end of 2015. In one of 34 provinces, there exists an integrated care model: the co-location of clinical psychologists in primary care clinics. This trial evaluates patient outcomes among those provided mental health care by GPs with those treated by clinical psychologists in primary care.

**Methods:**

In this partially-randomised, pragmatic, two-arm cluster non-inferiority trial, 14 primary care clinics were assigned to receive the WHO mhGAP training and 14 clinics with the co-location framework were assigned to the Specialist arm. Participants (patients) were blinded to the existence of the other pathway, and outcome assessors were blinded to group assignment.

All adult primary care patients who screened positive for psychiatric morbidity were eligible. GPs offered psychosocial and/or pharmacological interventions and Clinical Psychologists offered psychosocial interventions. The primary outcome was health and social functioning as measured by the HoNOS and secondary outcomes include disability measured by WHODAS 2.0, health-related quality of life measured by EQ‐5D-3L, and resource use and costs evaluated from a health services perspective, at six months.

**Results:**

153 patients completed the outcome assessment following GP care alongside 141 patients following Clinical Psychologists care. Outcomes of GP care were proven to be statistically not inferior to Clinical Psychologists in reducing symptoms of social and physical impairment, reducing disability, and improving health-related quality of life at six months. Economic analyses indicate lower costs and better outcomes in the Specialist arm and suggest a 50% probability of WHO mhGAP framework being cost-effective at the Indonesian willingness to pay threshold per QALY.

**Conclusion:**

General Practitioners supported by nurses in primary care clinics could effectively manage mild to moderate mental health issues commonly found among primary care patients. They provide non-stigmatising mental health care within community context, helping to reduce the mental health Treatment Gap.

**Trial registration:**

ClinicalTrials.gov NCT02700490

## Introduction

In many countries, low supply of mental health professionals and low demands for psychiatric services due to stigma, contribute to the wide Treatment Gap. While the median worldwide Treatment Gap for psychosis is 32.2% [1], the Treatment Gap in Low and Middle-Income Countries (LMICs) is estimated to be above 90% and reportedly 96.5% in remote parts of Indonesia [2]. Experts suggested integrating mental health care into primary care, to help bridge this gap [3]. Geography and health are intrinsically linked [4] as reflected in the adjustment of the health services to consider population density, demographic profile, and accessibility. The Indonesian government relies on a network of around 10,000 community health centres or *Pusat Kesehatan Masyarakat* (*Puskesmas)*, to provide residents with primary health services [5].

Recent research established that primary care clinics are the first port-of-call for most people with mental health problems [6]. Diagnosing mental health problems in primary care is difficult for several reasons. Firstly, most patients present with physical ailments [6, 7]. Secondly, underlying psychiatric morbidity may not be diagnosed given the time constraint during the consultation [8]. Patients may not be familiar with articulating their symptoms, and therefore the onus is on primary care doctors to provide a thorough clinical interview, which may be time-consuming. Thirdly, the majority of primary care doctors report that they could not refer patients on for secondary care [9]. In addition, when referrals are made, no appointment is ever made for up to 90% of referrals made to offsite practitioners [6, 10, 11]. Despite the logistical challenges, managing mental health problems in primary care may be the way forward. Several studies have shown the provision of mental health services in primary care is both effective and efficient [9, 12, 13]. Additionally, systematic reviews have shown that non-specialist health worker interventions for mental health care in LMICs are clinically effective [14, 15].

The WHO Mental Health Gap Action Programme (WHO mhGAP) was launched in 2008. The programme aims to support countries in the scaling up of services for mental, neurological, and substance use disorders (MNS). Both the Intervention Guide and the Training Manual are freely available on the WHO website. In 2015, the Indonesian Ministry of Health adapted the programme as an add-on training to selected pairs of *Puskesmas* GPs and Nurses, with the aim to extend the training to all *Puskesmas* GPs and Nurses. There had been no attempt to evaluate the effectiveness of this programme in Indonesia, prior to this study.

While the first WHO mhGAP Intervention Guide has been used by over 80 countries and translated into more than 20 languages, few research studies had directly addressed its use in LMICs, highlighting the pressing need for evidence [16]. Studies on barriers and facilitators to mhGAP-IG use, adherence, and patient outcomes are particularly required, to inform local, regional, national, and global improvements. A recent systematic review of the WHO mhGAP evidence from LMICs underlined the pressing need for an understanding of contextual challenges in the field, detailed protocols, qualitative studies, as well as randomised controlled trials [17]. It underlines the dire need for trials like as described here.

On the other hand, integrating psychological services in primary care is in line with current thinking which conceptualises primary care as a biopsychosocial rather than a biomedical field [18]. The presence of Clinical Psychologists in *Puskesmas* (co-location) combines the underlying concepts of the Chronic Illness and Integrated Care models through facilitating shared decision making between primary care providers and behavioural care providers, addressing the realities of primary care [18]. An integrated care model in high income countries has been shown to be cost-effective [19], matches patients’ preference (especially older patients) [20], leads to increased utilisation of mental health care [21] and results in higher treatment adherence and better clinical outcomes [13].

In 2004, Indonesia initiated the integration of Clinical Psychologists into primary care [22]. By 2016, two pilot districts in Yogyakarta Province (43 clinics) had employed Clinical Psychologists to provide basic mental health services at community level. This programme continues to expand to other districts, being introduced to a third district in 2017. It is considered an important step towards scaling up mental health services [23]. There is good evidence for the effectiveness of psychotherapy delivered in primary care [23] and that psychotherapy is as effective as antidepressant medication [24]. The co-location of Clinical Psychologists in primary care improves collaboration and potentially reduce the stigma of mental illness and barriers to care [25–27].

There remain doubts whether GPs were able to effectively manage mental health problems, given the logistical challenges. Conversely, policy makers are mindful of the enormous costs of hiring a Clinical Psychologist for every primary care clinic. This trial aims to compare patient outcomes following primary care mental health treatment by GPs and Nurses additionally trained in the WHO mhGAP or a Clinical Psychologist co-located in a *Puskesmas*. The systematic introduction of the WHO mhGAP framework presented an opportunity for a pragmatic trial comparing the two approaches. The outcomes examined include mental and social health symptoms, disability rating, and health-related quality of life. Cost-effectiveness study examined health systems costs associated with patient improvements at six months after enrolment.

## Methods

### Study design

This study set out to compare patient outcomes following mental health care either by GPs and Nurses randomly selected to attend additional training in the WHO mhGAP or Clinical Psychologists co-located in primary care, through a partially randomised, pragmatic, two-arm cluster non-inferiority trial, which enabled an examination of patients derived from whole populations in a ‘real world’ setting [28].

The WHO mhGAP programme was a new intervention to be introduced to randomly selected *Puskesmas* in the province alongside the study, modifying subsequent service delivery in these primary care clinics. The systematic, province-by-province introduction of the WHO mhGAP programme to randomly selected *Puskesmas* with the intention of scaling up to all 10,000 provided a phenomenon allowing a natural experiment to be conducted. The presence of the Specialist co-location model in two out of five districts in Yogyakarta province allowed a rigorous comparison between the effectiveness of the two frameworks.

The trial involved two phases: a pilot study in June 2016 with the objectives to refine data collection procedures and to serve as a practice run of the logistics for clinicians involved in the trial; as well as a substantive study between December 2016 to June 2017. Trial protocol is appended as S1 File.

### Ethics and governance

Ethics approval for the study was granted by the University of Cambridge Psychology Research Ethics Committee (reference number PRE.2015.108) and Universitas Gadjah Mada (reference number 1237/SD/PL.03.07/IV/2016). Trial insurance further covers investigators and research participants (University of Cambridge Trial Insurance reference number 609/M/C/1510). Ethics approval from all the clusters was not required as each cluster (*Puskesmas*) is a state-owned clinic funded and managed by district governments. Permission to conduct research at the Province of Yogyakarta including its all five districts was obtained from the Provincial Government Office (reference number 070/REG/V/625/5/2016). Additional permits were also obtained from each of the five districts. This trial was registered with clinicaltrials.gov on 25 February 2016, NCT02700490.

### Interventions, adherence and fidelity

The Ministry of Health adapted the WHO mhGAP framework to complement primary care GPs and Nurses’ existing set of competencies for recognising and managing a comprehensive list of psychiatric conditions. In the WHO mhGAP Arm, *Puskesmas* GPs supported by Nurses provided pharmacological therapy and/or psychosocial intervention as per the WHO mhGAP Intervention Guide, and/or referred participants to specialist care, as they saw fit. As the trial was designed to reflect real-life practice, GPs’ choice of treatment was not recorded, nor their use of the adapted WHO mhGAP modules enforced.

In the Specialist Arm, Clinical Psychologists typically use a combination of basic and advanced psychosocial therapies to manage disorders. As the trial was designed to reflect real-life practice, *Puskesmas* psychologists’ choice of treatment for research participants was not recorded.

As the objective of the pragmatic trial is to compare patient outcomes following standard care in both treatment arms, and to avoid changes in behaviour because of observation (Hawthorne Effect), adherence to WHO mhGAP Intervention Guide or clinical care guidelines was not observed or enforced. The intention-to-treat (ITT) approach is applied to the design, conduct, and statistical analysis of the trial to avoid overoptimistic estimates of the efficacy and feasibility of an intervention [29]. It was impossible to meaningfully assess the prescription of medication during the treatment or the psychosocial treatment used, for several reasons:

1. The recording of prescription was inconsistent.
2. The availability of psychotropics medication across participating *Puskesmas* varied.
3. The group size for any given diagnosis ranged from 1 (for agoraphobia, Specialist arm) to 56 (mixed anxiety and depression, Specialist arm).

### Setting

Yogyakarta is a province in Java, the only place in Indonesia where the Specialist co-location framework was operational at the start of the study, in two of five districts (Sleman and Kota). There are no significant sociodemographic differences between the five districts [30, 31]. Thus, all five districts in the province were considered eligible for the WHO mhGAP training, while only two districts with existing Specialist Co-Location framework formed the population pool for the comparison arm.

All 121 *Puskesmas* in Yogyakarta had received International Organisation for Standardization (ISO) Certification, so that data collection points could be embedded within routine patient flow procedures. As in pragmatic trials, the interventions were delivered in normal practice, by clinicians with typical experience and relying on routine care pathway procedures [28]. The screening and recruitment procedure were identical across all trial sites.

Within the study design, the unit of allocation was the *Puskesmas*, and the unit of observation and analysis was the service user. The cluster model allowed service providers to adhere as a ‘whole service’ to the treatment they were trained to provide.

### Allocation, training, blinding

All 121 *Puskesmas* in the province were eligible to take part in the trial as they were receiving state funding and were operating a general adult clinic. The Ministry of Health training budget allowed for only 14 *Puskesmas* in Yogyakarta province to receive the first WHO mhGAP training for the province. Therefore, the size of treatment arms was set at 14 *Puskesmas*. The scheduling of training for the province was aligned to this trial. The *Puskesmas* were assigned to each treatment arm in a 1:1 ratio.

Selection of Puskesmas was conducted by the WHO Collaborating Centre for Research and Training in Mental Health and Service Evaluation in Verona (in January 2016). Stratified random sampling, based on the number of clinics per district and the population size of districts, was used to assign Puskesmas to treatment arms using a random number generator. As the Puskesmas assigned to the control arm only came from two of the five districts, this trial was partially randomised [32].

The allocation list was then handed over to the Ministry of Health, whereupon the 14 which were allocated to the WHO mhGAP arm received a letter of invitation by the Ministry of Health to participate in the training. The Directorate of Mental Health conducted the training in April 2016, covering all WHO mhGAP modules, plus a day of role-play and observations of ‘real world’ setting.

All Clinical Psychologists involved in the trial had professional registration as a psychologist in Indonesia. *Puskesmas* psychologists attend regular continuing professional development (CPD) training organised by the Centre for Public Mental Health, a collaborator of this trial.

All clinicians were current treatment providers in *Puskesmas* and were employees of the District Health Authorities. Therefore, they were not hired specifically for the study, rather the study was integrated into their work. Following a discussion with the Head of Medical Services of the Province in 2016, to ensure the sustainability of screening and treatment procedures beyond the duration of the trial, clinicians were not paid to participate in the trial. Before the commencement of the Substantive Study in December 2016, all clinicians involved in the trial attended a one-day training session on the study procedure and on structured psychiatric interviews in non-specialised health settings (CIS-R), led by an author of this paper (SGA).

The clustered nature of the study allowed patient-participants to be blind to the existence of the alternative care pathway. Clinicians were not blinded to the treatment arm allocation. Outcome assessments at follow-up were conducted during home visits, and by a different assessor (trained research assistant) blinded to treatment allocation.

### Patient recruitment

Recruitment took place between 1–24 December 2016.

Participants were adult primary care patients visiting any of the 28 *Puskesmas* during the recruitment period, and not currently on any psychosocial or pharmacological therapy for mental health issues, who met the screening criteria (GHQ-12 score of 2 or above, GHQ scoring method 0-0-1-1) [33]. Primary care attendees who self-referred to the psychology service were invited to participate if they did not have ongoing treatment for mental health issues. Those receiving ongoing treatment for mental health issues were excluded from participating in the trial.

All participants attended primary care with complaints of a physical ailment, and therefore all participants are likely to have mental and physical multimorbidity. Reflecting the patient population in real world setting, patients with multimorbidity are not excluded in keeping with the principles of a pragmatic trial [34]. In other countries, mental and physical multimorbidity was found to be common among older people [35], and conversely, physical ailments are common among psychiatric patients [36]. The WHO mhGAP protocol stated “Depression commonly occurs alongside other MNS conditions as well as physical conditions” [37, 38]. In this pragmatic trial, only chronic common conditions, diabetes and hypertension, were recorded.

Primary care patients were given the screening questionnaire at the registration counter, when they obtained a queue number, to be self-completed while waiting for routine blood pressure test. Research assistants trained in scoring the GHQ identified patients who met the screening criteria. Research assistants provided a brief overview of the trial and an information sheet. To ensure avoidance of any sense of coercion, participants were asked to provide written informed consent before their GP appointment and were also reassured that declining would not affect usual medical care. Participants were invited to ask questions for clarification. Research assistants double-checked potential participants’ understanding of the follow-up requirements and their rights to withdraw prior to obtaining participants’ signature on the consent form. Those who consented were given a questionnaire booklet containing the outcome measures to complete while waiting to meet either the GP or Clinical Psychologist, depending on which cluster they were at. Participants were also informed that they were free to withdraw at any time during the study. There were procedures in place to assist illiterate patients, which were not required during the recruitment period of this study.

In the WHO mhGAP treatment arm, participants’ medical records and questionnaire booklets were passed to the GP who had received the add-on mental health training. Standard medical consultations took place, followed by a structured interview comprising the CIS-R and HoNOS, located in the second half of the questionnaire booklet. GPs would then record participants’ names, medical record number, and contact phone number or home address separately. Plans for medications and/or psychosocial therapy, if deemed necessary, were developed together with participants and they were asked to return for further therapy sessions.

In the Specialist arm, participants’ medical records were passed to a GP along with a request for a referral to a psychologist. Standard medical consultations for any physical ailments took place before participants went to a psychology consultation room for a structured interview with the Clinical Psychologist. The Clinical Psychologist then completed both the CIS-R and HoNOS, located in the second half of the questionnaire booklet. Participants’ names, medical record number, and contact phone number or home address would be recorded separately. Participants were asked to return for further therapy sessions with only the Clinical Psychologist, based on their needs.

The questionnaire booklet incorporated a section where the clinician could indicate participant’s diagnosis at the end of the in-depth interview. This diagnosis was not determined by the CIS-R score, but rather was based on the clinical judgment of each GP or Clinical Psychologist. This diagnosis determines whether participants were asked to return for intervention sessions.

### Follow-up assessment

Only participants with a diagnosis were contacted six months later for a follow-up assessment. Trained and vetted research assistants blinded to treatment arm allocation conducted the follow-up interview. They contacted participants from their original primary care clinic via telephone approximately a month to a fortnight before follow-up home visit. Participant personal details including phone numbers were not kept or shared. Participants were re-assessed using the full battery of questionnaires. At this point, no additional/replacement diagnosis was assigned to the participants.

### Measures

#### Screening

The twelve-item General Health Questionnaire (GHQ-12) was developed to screen for general (non-psychotic) psychological morbidity among primary care patients (Goldberg & Williams, 1988). Items on the GHQ-12 are rated on a 4-point scale using a time-frame of ‘in the last two weeks.’ There are two ways of scoring the GHQ-12: the bimodal GHQ scoring method (0-0-1-1) recommended by the test authors for use in clinical settings; and the Likert scoring method (0-1-2-3) which is commonly used in research settings. As this pragmatic trial took place in a clinical setting, the bimodal scoring method was used, and patients with score 2 and above were invited to participate in the study.

The screening questionnaire incorporates additional demographic questions on gender, education, employment, and utilisation of health services based on the Client Service Receipt Inventory (CSRI) [39].

#### Primary outcome measure

The Health of the Nation Outcome Scale (HoNOS) [40] is a 12-item scale to rate mental health service users in various aspects of mental and social health, each on a scale of 0–4. The ratings were made once all the information became available through clinical interview and took less than 5 minutes for the clinician to complete. HoNOS Total Score is a sum of individual item scores, with higher scores indicating greater mental or social health problems but was not an indication of the severity of the disorder. The primary outcomes were changes from baseline to the 6-month follow-up assessment in the health and social functioning of participants, as measured by the HoNOS [40].

#### Secondary outcome measures

Symptoms, disability, and quality of life are common key outcomes in pragmatic trials [28].

The WHO Disability Assessment Schedule (WHODAS 2.0) is a generic assessment instrument for health and disability used across all diseases, including mental, neurological, and addictive disorders. The WHODAS 2.0 covers six domains of functioning: Cognition; Mobility; Self-care; Getting along; Life activities; and Participation. This trial used the 36-item, self-administered version, taking approximately 20 minutes for self-completion. The complex scoring method based on item response theory was used, as advised by the WHO.

The 3-level European Quality of Life Scale (EQ-5D-3L) is a standardised self-report measure of health-related quality of life [41]. EQ-5D-3L ratings are commonly converted into composite utility scores using country-specific value sets, which measure people’s preferences in relation to health and weight each of the levels in each EQ-5D-3L dimension accordingly. The single utility scores represent the state of health-related quality of life for a person at any given time. As there is no Indonesian value set for the EQ-5D-3L, the existing Malaysian value set was deemed to be the closest alternative given the shared culture, predominant religion, and language of both countries (See S2 File).

Client Service Receipt Inventory (CSRI) by Beecham and Knapp (2001) is a generic questionnaire that records demographics information and health services utility within a specific period (e.g. 6 months). Data collected from the CSRI indicated the number of outpatient visits to *Puskesmas*, inpatient stay, and visits to the emergency.

For the evaluation of psychiatric morbidity, the clinician conducted a structured interview using the Revised Clinical Interview Schedule (CIS-R) [41]. The CIS-R is a fully structured diagnostic instrument that was developed from an existing instrument, revised and developed into a fully structured interview to increase standardisation and to make it suitable to be used by trained lay interviewers in assessing minor psychiatric morbidity in the community, general hospital, occupational and primary care research. In the CIS-R, there are 14 different symptom groups which participants were asked to consider, regarding the last month prior to the interview, focusing on symptoms experienced within the last week. Total CIS-R Scores are the sum of each symptom group with higher scores indicating higher levels of symptomatology.

As the CIS-R specifically diagnoses mood and anxiety disorders, participants with an indication of other disorders (psychosis, sleep disorders, dementia) were asked additional questions which enabled clinicians to establish an ICD-10 diagnosis. ICD-10 is widely used in the Indonesian mental health services as the national guideline for the diagnosis of psychiatric disorders (Pedoman Panduan Diagnosa Gangguan Jiwa) is the adapted version of ICD-10.

Clinician’s own diagnosis was recorded in participant’s interview booklet. While aided by the CIS-R interview questions to make a diagnosis, clinicians formed a diagnosis independently of CIS-R score. During the trial, clinicians were not provided the CIS-R diagnosis for their patients, which was based on its scoring algorithm. The CIS-R scoring took place during the data analysis.

The following were secondary outcomes:

1. Changes from baseline to the 6-month follow-up assessment in the disability of participants, as measured by WHO Disability Assessment Schedule 2.0 (WHODAS 2.0) [42].
2. Quality-adjusted life year at 6-month follow-up, as computed using the European Quality of Life Scale (EQ‐5D-3L) [43].

Attempts were made to collect reasons for treatment discontinuation and loss to follow-up.

### Translation and back-translation

The GHQ-12, HoNOS, WHODAS 2.0, and EQ-5D-3L in Bahasa Indonesia existed prior to the conception of this trial. The Bahasa Indonesia version of the GHQ-12, HoNOS, and WHODAS 2.0 were obtained from the National Institute of Health Research and Development, Ministry of Health, Indonesia. The Bahasa Indonesia version of the EQ-5D-3L was obtained from the Euroqol Group. The CIS-R was translated into Bahasa Indonesia by an author of this study (SGA), and back-translated into English by an Indonesian clinical psychologist.

### Sample size calculation

First, the sample size for non-inferiority individual randomisation RCT was calculated as a reference, using the estimated mean total HoNOS rating as a primary outcome [40]. Assuming a statistical significance value (α) of 0.05 and a statistical power of 0.80, a standard deviation of 5.2, and a clinical significance threshold (δ_0_) of 2 [44], the minimum sample size required was 84.

A larger sample size was needed to compensate for the clustering effect. Our approach was simplified because it did not consider variations in the number of participants in each cluster. Although this type of imbalance in cluster size may reduce the power of the trial, the loss is negligible for studies with more than 100 patients per arm [45]. Based on the additional assumption of an ICC of 0.1, the number of patients required would be 189 in each arm. Implementation research studies showed that in medical settings ICCs for outcome variables were generally lower than 0.05 [46]. In this trial, we decided to assume a high value for the ICC to consider a possible wide variation across different *Puskesmas*.

With an attrition rate of approximately 20%, we expected that a sample size of about 227 patients per treatment arm (approximately 16 for each *Puskesmas*) should yield sufficient power. Following the completion of baseline recruitment, a more accurate estimate of ICC and therefore the required sample size have been re-calculated (See S3 File).

### Analysis

#### Intervention uptake

Participant engagement with the treatment process was summarised and reported descriptively. There was no consensus regarding the appropriate number of therapy follow-ups (dose).

#### Analysis of primary and secondary outcome measures

These analyses were carried out using the STATA software package, version 13. Cleaning of outcome and baseline data was conducted without the treatment group allocations in view. Summary statistics from these preliminary analyses were reviewed to identify data errors.

Preliminary analyses compared the characteristics of participants with and without complete data at six-months follow-up, by treatment group. They were carried out for the primary and secondary outcomes. This analysis was used to develop an understanding of the missing data mechanism and to determine the appropriate methods for dealing with missing outcome data.

The unit of allocation was the clinic unit, and the unit of observation and analysis was the service users. The objective of conducting a non-inferiority trial was to demonstrate that neither the WHO mhGAP framework nor the Specialist framework was worse than the other with regards to clinical outcomes such as symptom severity and wellbeing.

Analyses were conducted using an intention-to-treat (ITT) approach. The effect of the type of service on HoNOS, EQ-5D-3L and WHODAS 2.0 scores at six months were analysed separately in mixed-effects random regression models. Considering the trial design, in which patients (level 1) were nested within clinic (level 2), the individual clinic was included in the models as a random effect. Each model included treatment allocation and the baseline score of the outcome investigated as fixed effects.

In secondary analyses, missing data on follow-up outcomes were estimated using multiple imputation by chained equations (MICE), which generated several different plausible imputed data sets and combined results from each of them. Predictive mean matching was used to deal with non-normality when imputing continuous variables. The robustness of the results with respect to violation of Normality was guaranteed by estimating CIs and p-values from 1000 bootstrap samples, using the non-parametric method.

The economic evaluation took a health systems perspective, in line with the effort from national and provincial governments to provide universal health coverage to citizens, and included the use of mental health resources including primary care clinic follow-up services. For all participants, service use was recorded using a modified version of the CSRI. At the follow-up, participants were asked to recall their use of health resources during the 6-month period. Differences in use of services between trial arms were compared and are reported for each service as the proportion of the group who had at least one contact. Statistical comparisons were not made to avoid problems of multiple testing and to keep the focus of the evaluation on costs and cost-effectiveness.

For each participant, a provincial unit cost was applied to each item of service use reported during the trial (at the follow-up interview) to calculate the total cost for the six months. Details of unit costs applied based on 2012 valuations of the costs of medical services in the province [47], published inflation rates [48], and retribution for infrastructure, ancillary workforce, and medical administration [49] are presented in online supplementary material (S4 File). Discounting was not relevant as the follow-up did not exceed 12 months.

The primary economic evaluation explored cost-effectiveness in terms of HoNOS, the primary outcome for the trial. A secondary cost-utility analysis explored effectiveness in terms of Quality-Adjusted Life Years (QALYs).

EQ-5D-3L utility scores were used to calculate QALY improvements during the 6-month period using the area under the curve approach [50]. Incremental cost-effectiveness ratios (ICERS), i.e. the additional cost of one intervention compared with another divided by the additional effect, were calculated based on parameter estimates from random-effects linear regression models that represent costs and both outcomes and take into account the clustered structure of these data. The uncertainty of these estimates was explored first, by bootstrapping 1000 resamples to generate a new distribution of estimates and plotting these onto a cost-effectiveness plane for interpretation and then, by constructing cost-effectiveness acceptability curves (CEAC). The CEAC is a plot of the probability of the intervention being cost-effective (y-axis) for a range of willingness to pay thresholds per unit improvement in outcome (x-axis) [51]. Initially, for the cost-utility analysis the WHO recommendation of three times GDP per capita [52, 53] or a calculation based on estimates of opportunity costs [54] were considered to approximate an Indonesian willingness-to-pay threshold, but recent research on the Indonesian willingness-to-pay threshold for medical intervention enabled a more exact analysis [55].

## Results

### Clinicians’ characteristics

Of the 14 GPs who took part in the trial, 3 were men while the rest were women. All GPs had civil servant status and received their licence to practise from the District government which manages the health budget for the district, including the management of all state-owned primary care clinics. All GPs were full-time permanent staff, and not on rotation, ensuring they stay within each clinic even after the end of the trial period. In comparison, all 14 Clinical Psychologists who took part in the study were women, and all were employed directly by the clinic. One Clinical Psychologist had to be dropped from the trial due to administrative issues.

### Patient recruitment

**Fig 1:**
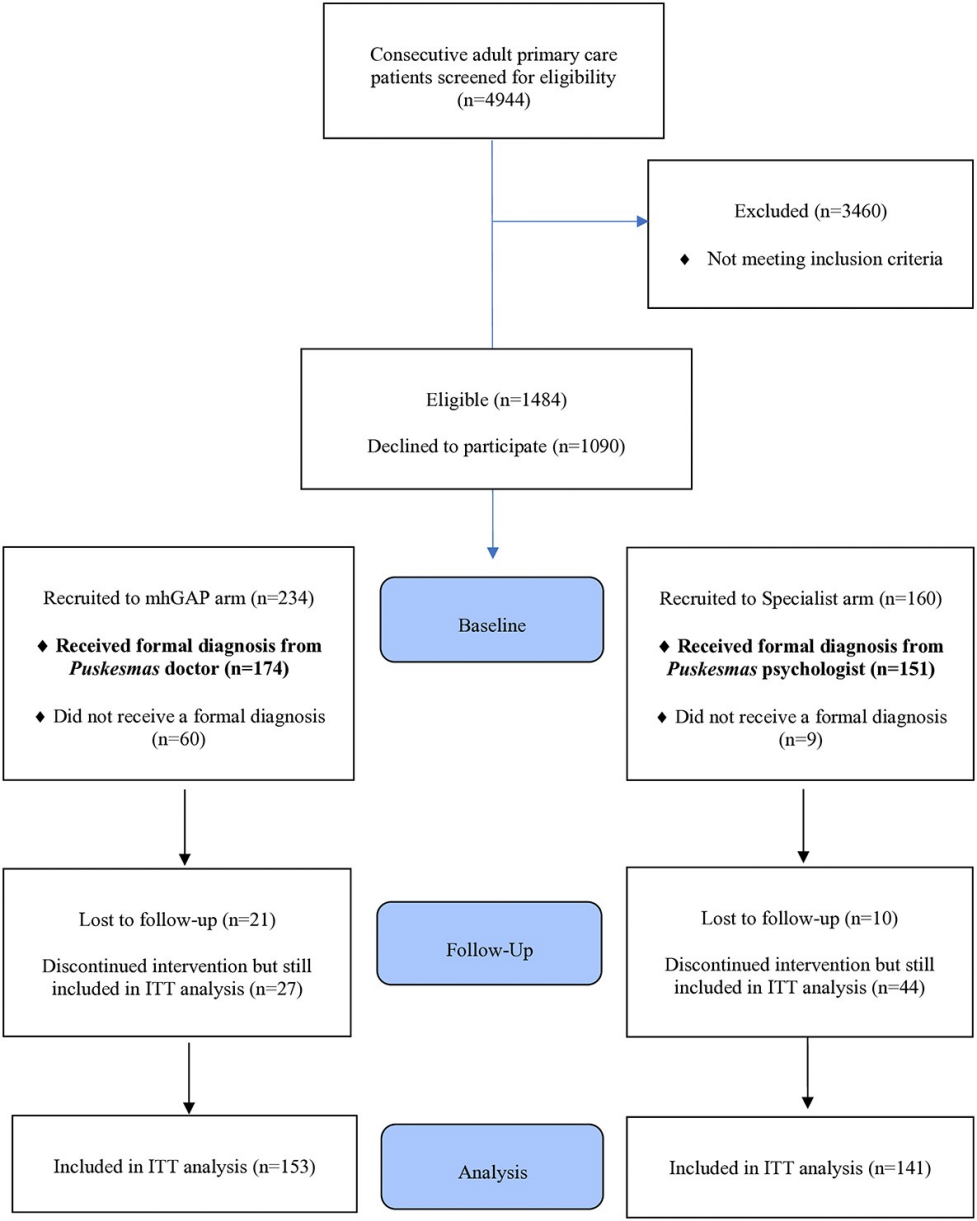
Participant flow diagram.

Trial participants were recruited from among adult primary care attendees at 27 participating *Puskesmas* (all 14 WHO mhGAP arm, and 13 Specialist arm). Recruitment took place between 1–24 December 2016. During the recruitment period, 4944 adult primary care patients attended the general medical clinic at 27 participating clinics (Fig 1). Following screening (n = 1484 met the screening criteria) and in-depth psychiatric interview (n = 394), 174 WHO mhGAP arm and 151 Specialist arm participants were given a formal diagnosis and recruited into the study.

### Revised sample size

The original target sample size was 189 per treatment arm, to account for possible clustering effect and an imbalance in the number of participants per cluster. Following the completion of the baseline measurements, the required sample size was therefore recalculated with an estimate of intra-cluster correlation of 0.025 for the primary outcome (HoNOS) derived from the baseline dataset. As the intra-cluster correlation coefficient was lower than the hypothesised one, a significant difference for the variable under investigation was detected with a minimum of 96 patients per arm. The formula is described in S3 File [56].

### Retention

While 325 participants in total were given a confirmed or probable diagnosis, and eligible for intervention, of these, 295 participants could be approached for re-interview at six months. Our retention rate is therefore 90.8%.

There was a good follow-up rate with more than three quarters attending one or more intervention sessions after baseline (223/295, 76%). We further assessed if the likelihood for returning for intervention is different among the two treatment arms. The number of participants returning for GP-conducted intervention is significantly more than to Clinical Psychologists (χ^2^ = 7.364, p = 0.007), 82% (n = 126) and 69% (n = 97) respectively.

### Participants lost to follow-up

At follow-up, 31 (9.7%) participants dropped out: 21 (12.1%) and 10 (6.8%) in the WHO mhGAP and Specialist arms, respectively (Participant Flow Diagram, Fig 1). Of these, 23 (74.2%) were women and 8 (25.8%) men, of mean age 43.4 (17.5) and median of 46 (30). More than half (n = 17, 54.8%) were of low education level, compared to 50% and 32% patients followed up from the WHO mhGAP and Specialist arms respectively. Considerably more participants lost to follow-up were unemployed at baseline (25.8%) compared to 10.3% patients followed-up at both the WHO mhGAP and Specialist arms. There were no notable differences in clinical characteristics at baseline with those who were followed-up.

Many participants did not have a current contact number retained at the primary care clinic. For these, a visit at their primary address was made. This task was made difficult by the lack of consistency in the recorded address, i.e. at times the recorded address detailed the village name, and not a home address. It was discovered that some participants had relocated away from their recorded address. As such, it was nearly impossible to track their whereabouts. Two participants have moved to a different province and did not attend the follow-up sessions. Five participants from a clinic were recruited to a different research project. For these five, their medical records had been removed from the clinic illegally. These participants could not be traced.

### Patient characteristics

At baseline, 174 WHO mhGAP arm and 151 Specialist arm participants received a formal diagnosis of psychiatric disorders. These were the participants invited to return for intervention and follow-up assessment six months after recruitment.

The groups did not differ in many socio-demographic characteristics, except for specialist arm patients, who were younger, with a higher educational level and a higher percentage of Indonesian speakers (Table 1). A sizeable proportion of participants from both treatment arms were diagnosed with Mixed Anxiety and Depression, 25.4% and 37.2% in the WHO mhGAP and Specialist arms respectively.

**Table 1:** Socio-demographics and diagnoses of patients assessed at baseline (n = 325).

	WHO mhGAP arm (n = 174)	Specialist arm (n = 151)	Test and Sig of Difference
**Gender, n (%)**			
Male	46 (26.4)	44 (29.3)	χ^2^ = 0.34, df = 1, p = 0.562
Female	128 (73.6)	106 (70.7)	
**Age**	(3 missing)	(1 missing)	
mean (SD)	44.1 (15.0)	38.5 (14.8)	t = 3.35, df = 319, p = 0.001
median (IQR)	46.0 (23.0)	38.0 (26.0)	
**Marital Status, n (%)**		(1 missing)	
Unmarried	34 (19.5)	45 (30.0)	χ^2^ = 4.98, df = 2, p = 0.083
Married	124 (71.3)	91 (60.7)	
Separated, Divorced, Widowed	16 (9.2)	14 (9.3)	
**Primary Language, n (%)**		(4 missing)	
Indonesian	16 (9.2)	27 (18.4)	χ^2^ = 9.21, df = 2, p = 0.010
Javanese (fluent in Indonesian)	142 (81.6)	115 (78.2)	
Javanese (not fluent in Indonesian)	16 (9.2)	5 (3.4)	
**Educational Level, n (%)**		(1 missing)	
Low (Elementary-Middle School)	86 (49.4)	46 (30.7)	χ^2^ = 11.74, df = 1, p = 0.001
High (High School, Diploma, Degree)	88 (50.6)	104 (69.3)	
**Living Condition, n (%)**	(1 missing)	(2 missing)	
Alone	19 (11.0)	22 (14.8)	χ^2^ = 2.43, df = 3, p = 0.487
Partner	93 (53.8)	69 (46.3)	
Relatives	21 (12.1)	17 (11.4)	
Parents	40 (23.1)	41 (27.5)	
**Working Status, n (%)**		(1 missing)	
Employed	84 (48.3)	75 (50.0)	χ^2^ = 0.45, df = 2, p = 0.800
Unemployed	24 (13.8)	17 (11.3)	
Housewife, Student, Retired, Volunteer	66 (37.9)	58 (38.7)	
**CIS-R Diagnosis at Baseline, n (%)**			
Depressive Episode	34 (19.7)	20 (13.5)	
Panic Disorder	17 (9.8)	3 (2.0)	
Obsessive Compulsive Disorder	0 (0.0)	3 (2.0)	
Social Phobia	16 (9.2)	2 (1.4)	
Agora Phobia	0 (0.0)	1 (0.7)	
Specific (Isolated) Phobia	6 (3.5)	2 (1.4)	
Generalized Anxiety Disorder	16 (9.2)	8 (5.4)	
Mixed Anxiety Depression	44 (25.4)	56 (37.2)	
Non-CIS-R Diagnoses given	40 (25.1)	54 (36.5)	

Table 2 captures a comparison of participants’ means of dependent variables at baseline and follow-up, as well as a test of significant difference. All total scores (dependent variables) were treated as continuous variables and a paired samples T-test was performed for each baseline and follow-up pair of scores. There is no significant difference between the two treatment arms at baseline for the severity of clinical symptoms (CIS-R Total Score), disability rating (WHODAS 2.0), and quality of life (EQ-5D-3L). The difference in HoNOS Total Score at Baseline is significant t = -2.40, df = 293, p = 0.039.

**Table 2:** Means of dependent variables at baseline and 6-months follow-up for participants who completed the trial.

	Baseline	Follow-up	Test and Sig of Difference
**CIS-R Total Score**			
Overall (n = 294)	10.97 (11.47)	4.43 (5.49)	t = 9.63, df = 293, p = 0.000
WHO mhGAP (n = 153)	10.08 (12.36)	4.63 (5.73)	t = 5.09, df = 152, p = 0.000
Specialist (n = 141)	11.93 (10.37)	4.21 (5.22)	t = 9.62, df = 140, p = 0.000
**HoNOS Total Score**			
Overall (n = 287)	5.45 (5.13)	3.45 (4.49)	t = 6.10, df = 286, p = 0.000
WHO mhGAP (n = 150)	4.64 (4.50)	3.40 (4.38)	t = 2.66, df = 149, p = 0.009
Specialist (n = 137)	6.34 (5.70)	3.50 (4.62)	t = 6.26, df = 136, p = 0.000
**WHODAS 2.0**			
Overall (n = 294)	23.62 (16.32)	12.41 (13.99)	t = 11.56, df = 293, p = 0.000
WHO mhGAP (n = 153)	24.23 (16.89)	12.10 (13.99)	t = 8.41, df = 152, p = 0.000
Specialist (n = 141)	22.95 (15.71)	12.77 (14.03)	t = 7.98, df = 140, p = 0.000
**EQ-5D-3L Malaysian Value**			
Overall (n = 295)	0.790 (0.132)	0.829 (0.113)	t = -11.01, df = 294, p = 0.000
WHO mhGAP (n = 153)	0.781 (0.135)	0.825 (0.112)	t = -9.32, df = 152, p = 0.000
Specialist (n = 141)	0.800 (0.128)	0.832 (0.115)	t = -6.31, df = 140, p = 0.000

### Drop-outs

During the 6-month period, 31 (9.5%) participants recruited at baseline declined to participate further in the trial or were untraceable. Of these, 21 (67.7%) belonged to the WHO mhGAP arm and 10 (32.3%) belonged to the Specialist arm. Most drop-outs were women (n = 23, 74.4%). More than half (n = 17, 54.8%) were married, and about half (n = 15, 48.4%) were living with their partners. More than half (n = 17, 54.8%) were educated below High School (low education), a greater percentage than those who completed the study.

### Primary outcome

Both groups experienced a similar improvement in the health and social functioning (HoNOS total score) at the 6-month follow-up: the regression coefficient of Specialist vs WHO mhGAP was -0.55 (95%CI -1.69 to 0.58) with p = 0.341 (Table 3). The bootstrap procedure gave 95%CI -1.46 to 0.35 with p = 0.232. The re-analysis by using one-sided 97.5%CI showed that for Beta≤0 p = 0.829. Multiple imputation analysis (31 observations imputed at follow-up) supported the result: the regression coefficient of Specialist vs WHO mhGAP was -0.55 (95%CI -1.67 to 0.56) with p = 0.333. The non-inferiority for GP-provided primary mental health care versus a clinical psychologist co-located in primary care was met for the primary outcome of HoNOS total score, with the upper bound of the 95% CI for difference between treatments not more than or equal to 2.

**Table 3:** Means (SDs) and mixed-effects random regression coefficients of Specialist vs WHO mhGAP (95% CI) for primary (HoNOS) and secondary outcomes (EQ-5D and WHODAS 2.0) of patients assessed at baseline (BL) and 6-month follow-up (FU), WHO mhGAP arm n = 152, Specialist arm n = 138.

PRIMARY OUTCOME	WHO mhGAP arm		Specialist arm		Regression coefficient^#^ of Specialist vs WHO mhGAP (95% CI)	p-value
	BL (n = 173)	FU (n = 152)	BL (n = 148)	FU (n = 138)		
HoNOS TOTAL	4.96 (4.76)	3.46 (4.40)	6.47 (5.70)	3.43 (4.61)	-0.55 (-1.69 to 0.58)^§^	0.341^§^
SECONDARY OUTCOMES	WHO mhGAP arm		Specialist arm		Regression coefficient^#^ of Specialist vs WHO mhGAP (95% CI)	p-value
	BL (n = 173)	FU (n = 152)	BL (n = 148)	FU (n = 138)		
EQ-5D-3L Utility Score	0.77 (0.14)	0.83 (0.11)	0.80 (0.13)	0.83 (0.11)	-0.01 (-0.02 to 0.01)	0.296
WHODAS 2.0 Total	25.82 (17.53)	(1 missing) 12.16 (14.04)	23.30 (15.85)	12.53 (13.98)	0.29 (-4.38 to 4.96)	0.903

^#^ Random effects linear regression models with *Puskesmas* as a random effect and the corresponding baseline score and the treatment assignment as fixed effects

Multiple Imputation with 20 replications:

HoNOS (31 imputed observations at follow-up) Beta = -0.55, 95%CI (-1.67 to 0.56), p = 0.333

EQ-5D (30 imputed observations at follow-up) Beta = -0.01, 95%CI (-0.02 to 0.01), p = 0.187

WHODAS (31 imputed observations at follow-up) Beta = -0.02, 95%CI (-4.33 to 4.30), p = 0.994

### Secondary outcomes

A similar improvement was estimated in both groups for the quality of life (EQ-5D Utility Scored using the Malaysian Algorithm: the regression coefficient of Specialist vs WHO mhGAP was -0.01 (95%CI -0.02 to 0.01) with p = 0.296 (Table 3). The bootstrap procedure gave 95%CI -0.02 to 0.01 with p = 0.312. The re-analysis by using one-sided 97.5%CI showed that for Beta≤0 p = 0.852. Multiple imputation analysis (30 observations imputed at follow-up) supported the result: the regression coefficient of Specialist vs WHO mhGAP was -0.01 (95%CI -0.02 to 0.01) with p = 0.187.

A similar amelioration was estimated in both groups for disability (WHODAS 2.0 total score) at follow-up: the regression coefficient of Specialist vs WHO mhGAP was 0.29 (95%CI -4.38 to 4.96) with p = 0.903 (Table 3). The bootstrap procedure gave 95%CI -2.52 to 3.10 with p = 0.840. The re-analysis by using one-sided 97.5%CI showed that for Beta>0, p = 0.548. Multiple imputation analysis (31 observations imputed at follow-up) supported the result: the regression coefficient of Specialist vs WHO mhGAP was -0.02 (95%CI -4.33 to 4.30) with p = 0.994.

Participants in the Specialist arm showed significantly higher HoNOS scores at Baseline (p = 0.010). The significant difference in baseline HoNOS scores were adjusted for in the regression modelling. Both treatment arms did not differ significantly in their baseline scores for the secondary outcomes.

### Recovery and remission

Among participants followed up from the WHO mhGAP arm (n = 173) and the Specialist arm (n = 151), a large proportion of participants were no longer meeting any diagnostic criteria according to CIS-R and were considered in remission (n = 152 and n = 134 respectively). There is no significant difference between the two arms on remission rate, Chi-square = 0.164, df = 1, p = 0.730.

Looking at the response to treatment among participants who had one or more intervention sessions, there was greater improvement in health and social functioning among the Specialist arm participants (mean reduction in HoNOS score of 2.86, SD 5.40) compared to WHO mhGAP arm participants (mean reduction in HoNOS score of 1.11, SD 5.82). This difference is statistically significant (t = 2.26, df = 214, p = 0.025). There is no correlation between the number of follow-up therapy sessions and the magnitude of reduction in HoNOS score, Pearson’s r = 0.04, p = 0.457.

### Economic analysis

A total of 153 participants in the WHO mhGAP arm and 140 in the Specialist arm were included in the economic analyses. A participant flow diagram for the economic analysis is presented in S1 Fig.

Table 4 below shows the use of inpatient services (number of overnight stays), outpatient medical care (number of appointments), as well as mental health intervention sessions at a primary care clinic (number of appointments). Participants did not report any visits to hospital emergency during the 6-month period. Due to the relatively easy access to specialist physicians, Indonesians seldom visit hospital emergency except for acute and severe situations, like traffic accidents.

**Table 4:** Average resource use by each treatment arm during the 6-month period.

	WHO mhGAP arm (n = 153)	Specialist arm (n = 141)
**Inpatient (number of nights)**		
n (%) of users	11 (7.2)	13 (9.2)
mean (sd)	0.59 (2.32)	1.25 (4.48)
maximum	13	26
**Outpatient (number of appointments)**		
n (%) of users	43 (28.1)	29 (20.6)
mean (sd)	0.90 (1.59)	0.89 (1.98)
maximum	5	10
**Intervention Session (number of appointments)**		
n (%) of users	126 (82.4)	97 (68.8)
mean (sd)	4.48 (3.39)	4.00 (3.53)
maximum	10	10

A greater proportion of participants returned for intervention sessions at the WHO mhGAP arm (82.4%) compared to the Specialist Arm (68.8%). The trial study was unable to capture the cost of prescribed medication due to lack of consistency regarding the provision of medication.

The table below details average costs over six months and outcomes for all participants who completed the assessments. Average HoNOS score at baseline was significantly higher among the Specialist arm participants (mean of 6.25, SD = 5.57) compared to WHO mhGAP arm (mean of 4.76, SD = 4.72), adjusted mean difference of -1.49. There was little difference in utility scores between the two arms and across follow-up, which resulted in small differences in QALYs between the two arms (Table 5).

**Table 5:** Mean outcomes per participant over the 6-month period.

	WHO mhGAP arm (I) N = 153	Specialist arm (C) N = 141	Unadjusted mean difference (I-C)	Adjusted mean difference (I-C)		
	Mean (SD)	Mean (SD)		estimate	95% CI	p-value
**Costs**						
Intervention	17481	30468	-12987			
MH Treatment	81145 (61377)	60388 (53253)	20807	20397	7163 to 33632	0.004
Total Cost	98626 (61377)	90856 (53253)	7770	7410	-5823 to 20645	0.2
**HoNOS Total**						
Baseline	4.76 (4.72)	6.25 (5.57)	-1.49	-1.41	-0.26 to 2.55	0.04
6-months	3.48 (4.39)	3.44 (4.59)	0.04	-0.1	-1.3 to 1.1	0.9
**Utility scores**						
Baseline	0.78 (0.14)	0.80 (0.13)				
6-months	0.83 (0.11)	0.83 (0.11)				
**QALYs**						
6-months	0.4015 (0.060)	0.4082 (0.059)	-0.0067	-0.0067	-0.02 to 0.007	0.6

For the primary cost-effectiveness analysis using HoNOS scores, the lower costs and better outcomes in the Specialist framework generate an ICER of Rp 4,843 per unit improvement in HoNOS score, which suggest that the Specialist framework may be better able to improve health and social functioning for the same cost as the WHO mhGAP framework.

The point at which the Specialist care has a higher probability of being cost-effective compared to the WHO mhGAP arm (i.e. probability >50%) is at a willingness to pay level of Rp 150,000 per unit improvement in HoNOS score.

For the secondary cost-utility analysis, the lower costs and slightly better outcomes in the Specialist framework generate an ICER of—Rp 11,105,970 per QALY which suggest that the Specialist framework dominates the intervention at the 6-month follow-up point.

The cost-utility analysis suggest that Specialist framework has a 50% probability of being cost-effective compared to WHO mhGAP framework at the Indonesian willingness to pay for medical interventions of Rp 11,000,000 per QALY. Full details of results of both economic analyses are provided in S5 File.

## Discussion

In this pragmatic trial comparing different delivery options for primary mental health care in a LMIC (Indonesia), several expected and unexpected outcomes were discovered.

This partially randomised, pragmatic, cluster trial explored two core aims:

1. the *clinical effectiveness* of the GP-provided primary mental health care versus care by a Clinical Psychologist co-located in primary care at six months
2. the *cost-effectiveness* of the the GP-provided primary mental health care versus care by a Clinical Psychologist co-located in primary care at six months.

GP-provided primary mental health care was shown to be statistically not inferior to care by a Clinical Psychologist co-located in primary care in reducing symptoms of social and physical impairment at six months. Moreover, no difference was found in disability and health-related quality of life during the same period. In both treatment arms, large proportions of participants went into remission. GP-provided primary mental health care had a higher percentage of patients returning for intervention.

The cost-effectiveness analysis using QALYs indicate the 50% probability of care by a Clinical Psychologist co-located in primary care being more cost-effective than GPs at the Indonesian willingness to pay for medical interventions.

Our results show that GPs were able to manage mental health problems in primary care and were likely the more preferred option resulting in a significantly greater proportion of patients returning for follow-up treatments. On the other hand, follow-up appointments with a Clinical Psychologist cost the health systems less, and as such may be a more cost-effective option in the long run.

### Strengths and weaknesses

The trial achieved its primary objectives of evaluating the clinical and cost-effectiveness of GP-provided primary mental health care versus care by a Clinical Psychologist co-located in primary care at six months. The trial is the first study which evaluates primary care mental health services in Indonesia. The systematic introduction of the adapted WHO mhGAP framework into the country’s primary care system allowed a robust trial to examine the impact of both frameworks in the Indonesian context, in a ‘real-life’ setting. The study showed a very high retention rate (90.4%) in the six months). It is also the first study which evaluates patient outcomes of care provided by non-specialists who had undergone the WHO mhGAP programme through a pragmatic trial design. As this is a pragmatic trial, efforts were made to reflect the ‘real life’ conditions as far as possible. The ITT approach was used in the analysis of the trial, reducing the potential for an overly optimistic estimate of the efficacy of both frameworks.

While it is beneficial to reflect on-the-ground lack of monitoring of treatment adherence it could also be considered a weakness, as there is no way of ensuring if the adapted WHO mhGAP training received by GPs had any contribution to the treatment outcomes of their patients. Future studies should attempt to incorporate this assessment. A third treatment arm comprising Puskesmas GPs who did not attend the WHO mhGAP training could have been considered.

While a pragmatic trial typically requires enormous budgets and a large manpower base, following the timeline of the Indonesian Ministry of Health and collaborating with Health Authorities at district and provincial levels, and matched-funded collaborators enabled significant reductions of resources required. The adapted WHO mhGAP “standardised” training was conducted by a dedicated team from the Ministry of Health, which was an advantage in terms of external validity, in that training was replicated in other provinces outside of the study, highlighting its pragmatic nature. Conducting a pragmatic trial without the backing of a large research grant from one principal funder was challenging, and it is no wonder that few people have attempted this. This study proved that it can be feasible and that the payoff is enormous. However, the length of the recruitment period and the overall size of the trial was a function of funding constraints.

We believe that the study design adequately addresses its main objectives, as well as able to inform clinical practice. It reflects what happens outside laboratory conditions, when GPs employed in state-run primary care clinics were provided an additional training to treat their patients. The trial could not impose any control of what kind of intervention each GP provided their heterogeneous patients with but was more concerned instead with the impact of feasibility of the management of mental health in primary care.

While the pragmatic nature is an advantage, the size of recruitment and delivery of intervention do have disadvantages. As with many mental health trials, there will always be concerns about the potential bias in the response of those patients who agreed to participate in research. Trial participants in the Specialist arm had a significantly higher average health and social impairment, as measured by HoNOS, compared to participants in the WHO mhGAP arm. While this may indicate a self-selecting bias, there are no significant difference across the two treatment arms in all secondary outcome measures. The nature of the recruitment process which we agreed on with the health services did mean it was impossible to estimate the demographic profiles of those who refused to participate. The recruitment procedure, unfortunately, created a long waiting time in clinics for those who agreed to participate. As such, there were inevitably dropouts even before the recruitment interview took place.

A significant issue with the economic evaluation pertains to the reliance on patient recall and report of health services utilisation, which for many reasons may be under- or over-reported. Moreover, the lack of Indonesian utility indices for EQ-5D-3L resulted in a reliance on the Malaysian indices. While the Malaysian utility indices were chosen due to higher proximity in culture and religion to Indonesia, it may not reflect the Indonesian preferences.

The cost-utility analysis used a willingness-to-pay (WTP) threshold of Rp 11,000,000.00 per QALY for medical intervention in the country [55], which was determined using a pay-for-service perspective, i.e. when patients must pay for interventions out of pocket. There is a lack of established Indonesian WTP threshold for medical interventions from the health systems perspective. What individuals are willing to pay out of pocket is likely to differ from a structural, top-down perspective of the country’s universal health coverage system. As this study aims to assist the Indonesian Ministry of Health in the scale-up of the most appropriate primary mental health service framework, the lack of established WTP threshold from the health systems perspective is significant.

Supported by adequate sample size for the chosen methodology [56, 57] our findings support existing literature in that the primary outcome, HoNOS, was able to detect change in the community for those with common mental disorders [58] and those with higher HoNOS scores at the diagnosis [59].

The culture of rent-seeking and bribery (or even discrimination) embedded within the primary care system resulted in inconsistent buy-in among gatekeepers (Heads of Clinics). One gatekeeper sought bribes, rejected screening for the first few days of recruitment, and placed the Clinical Psychologist at this clinic on leave for the remaining duration of recruitment. As a result, the cluster was dropped out of the trial.

### Possible mechanisms

As hypothesised, GPs were shown to be as effective as Clinical Psychologists in providing mental health care at primary care level. This result should be viewed keeping in mind that most participants had mild to moderate severity of disorders, in line with the findings of existing studies. Recovery and remission could therefore be attributed to the natural course of the disease rather than the effectiveness of treatment. Relatively brief psychosocial therapy was perhaps sufficient, and follow-up sessions could perform a monitoring function to ensure patients were not getting worse. The Clinical Psychologists started out with patients of higher morbidity ratings (more severe cases) but could reduce the clinical symptoms to remission similar to GP patients.

There is a possible selection effect for HoNOS and clinical rating scores (CIS-R) as evinced by the significantly higher average scores of participants in the Specialist arm, compared to the average scores in the WHO mhGAP arm. While the reason for this is unclear, we hypothesise that primary care attendees who agree to see a Clinical Psychologist might be those with clearer impairment in health and social functioning, i.e. those who saw the need for specialist care. From existing literature, we are aware that insight into one’s challenges is a determinant in help-seeking among those with mental health issues. This indicates that there is still a stigma associated with specialist mental health care. If this hypothesis is erroneous, our findings indicate that the randomisation procedure performed well for the secondary outcome measures, but not HoNOS. From a statistical perspective, this does not change our results, as the difference was adjusted for in the regression modelling.

The percentage of participants returning to intervention by Clinical Psychologists were markedly lower than those returning to GP care. It is possible that participants did not return for intervention due to stigma, misconceptions, or other reasons. In-depth qualitative research needs to shed light on this. The low return rate would not be a surprise to clinicians involved. Clinical Psychologists involved in the trial mentioned during a training session that in general, few patients returned for intervention. There is potentially a limited understanding among patients that psychological intervention requires several sessions. While the idea of a ‘one-session fix’ could be attributed to the lack of communication regarding treatment plans with patients, there are indications that psychologists seldom develop treatment plans alongside their patients. Further studies on shared decision-making could potentially resolve this issue.

Regarding cost-effectiveness, the Specialist Arm had on average lower costs for intervention because of two possible reasons. Firstly, the average hourly cost of Clinical Psychologists is lower than that of GPs. Secondly, there are fewer follow-up appointments made with a Clinical Psychologist (97 recorded sessions) compared to GPs (126 recorded sessions). Despite the higher cost of the initial appointment, i.e. to receive free psychology consultations, a patient needs GP referral, overall aggregate of treatment cost (initial interview and intervention combined) was slightly lower than GP care. Coupled with more substantial improvements in health outcomes, Clinical Psychologists may be the more cost-effective option.

While the Specialist arm clinics had more patients who screened positive for psychiatric morbidity, many more patients declined participation in the trial. It is possible that there is a stronger stigma associated with a mental health consultation with a psychologist than the generic nature of GP consultations. Stigma has been found to be a barrier to help-seeking [60] resulting in low demands for mental health services. Seeking help from a Clinical Psychologist requires recognition of one’s mental health needs. In comparison, seeking mental health assistance from a GP, especially one that the patient already holds a queue number for, may be deemed more palatable. Separating mental health care from physical health care can entrench isolation and encourage stigma towards those affected [61, 62]. The provision of mental health care by GPs addresses this isolation and stigma issue to a large extent.

### Comparison with existing literature

While the first WHO mhGAP Intervention Guide was used by over 80 countries and translated into more than 20 languages, few research studies had directly addressed the use of the mhGAP framework in LMICs, highlighting the pressing need for evidence [16]. A recent systematic review of the WHO mhGAP evidence from LMICs found 13 studies describing the training of health workers using the WHO mhGAP Intervention Guide but only nine studies describing the clinical implementation of the WHO mhGAP framework in LMICs [17]:

1. In **Ethiopia**, a survey of experiences, strengths, and challenges of integrating mental health in primary health centres was conducted [63].
2. Charlotte Hanlon and team (2016) proposed a randomised, controlled, non-inferiority trial based on task-sharing model also in **Ethiopia**, where health centre nurses and health officers were trained to deliver mental health care for people with severe mental disorders, based on the WHO mhGAP framework [64].
3. In **Haiti**, a retrospective chart review of outpatient assessments using the WHO mhGAP framework was conducted [65].
4. A mixed methods study of acceptability and patient satisfaction of mental health care plan designed by staff trained using the WHO mhGAP framework was conducted in **Nepal** but did not examine clinical outcomes [66].
5. A study in **Afghanistan** examined the functionality and acceptability of a WHO mhGAP mobile application used by primary health centre clinicians but did not consider patient outcomes [67].
6. Several studies in **Kenya**, including an evaluation of a WHO mhGAP mobile application for depression screening and a longitudinal non-randomised interventional study of adult patients seeking care for depression from rural public healthcare workers or traditional health practitioners trained in WHO mhGAP framework were conducted [68–70]. Over three months, patients who sought care from traditional health workers were found to have significant reductions in depression symptoms as measured by the Beck Depression Inventory.
7. In **Zambia**, an RCT of WHO mhGAP framework intervention for alcohol problems with an 8-week follow-up was conducted at a hospital in Lusaka [71]. The intervention group was found to have a significantly longer abstinence period.

Studies by Musyimin et al (2017a) in Kenya and Sheikh et al (2017) in Zambia were the only two existing literature reporting patient outcomes for interventions conducted with the WHO mhGAP framework. Studies proposed by Hanlon et al (2016) to take place in Ethiopia and conducted by Sheikh et al (2017) in Zambia were the only two existing literature reporting a robust experimental design. The pragmatic trial reported here is hitherto the only completed evaluation of an adapted WHO mhGAP framework looking at patient outcomes with robust experimental design taking place in real primary care setting.

On the other hand, literature evaluating the effectiveness of collaborative co-location of psychologists in primary care is even more limited, despite the abundance of articles extolling the virtues of a collaborative care model. Looking at the co-location of psychologists in primary care, a report shows between 14% (South Dakota) to 50% (Rhode Island) GPs sharing practice with a psychologist in American states [72]. The same report stated a positive correlation between the state supply of psychologists and the percentage of co-location (Pearson’s r = 0.58). A meta-analysis has shown that integrated care model improves patient outcomes, although it is unclear whether co-location is either necessary or sufficient for improving outcomes [73]. This pragmatic trial is therefore the only completed evaluation of ‘real world’ co-location framework in Indonesia, and among the first worldwide, looking at patient outcomes, with robust experimental design.

That our participants were more amenable to return to a GP than a Clinical Psychologist is a stark contrast to a previous study of patient preferences for mental health service providers conducted in England alongside an effectiveness trial [74]. The English study found that when given a choice, primary care patients who met the criteria for depression preferred non-directive counselling or cognitive behaviour therapy with a psychologist instead of GP care. Primary care patients were also reluctant to risk being randomly allocated to GP care. More in-depth, context-specific and cross-contextual research should be undertaken to understand these differences fully.

### Implications

While the adaptation of the WHO mhGAP in Indonesia highlighted the government commitment to mental health care, without a monitoring system to ensure accountability, treatment adherence is unknown, and efficacy not guaranteed. Without a monitoring system in place, perhaps the health system should focus on introducing a screening procedure to assist with the identification of psychiatric morbidity.

Arguably, all Indonesian medical graduates would have competency in diagnosing and (at least) initial intervention of all ICD-10 psychiatric diagnoses commonly found in primary care. GPs should therefore be able to recognise and manage these disorders in the first instance, or have a procedure for referral otherwise. Moreover, without a screening procedure as implemented in this trial, GPs may not recognise or want to spend the time uncovering patients’ psychological needs. This leads to questions regarding the value proposition of the adapted WHO mhGAP framework in the Indonesian context. As the implementation cost is enormous, the Indonesian Ministry of Health should wisely reconsider the nation-wide scale-up and resist international pressure to take action. The WHO mhGAP framework, despite its adaptation, is a costly exercise which may not be the answer the country was hoping for. Without the WHO mhGAP training, however, GPs are reluctant to provide psychosocial intervention to their patients. Perhaps a primary focus on screening and early identification, followed by initial intervention by publicly trusted GPs, relying on their existing medical competencies, is a less costly and feasible alternative. A second focus on strengthening the referral system for moderate to severe cases, may help reduce the mental health Treatment Gap.

There is a significant need for all clinicians to discuss treatment plans with patients and for patients to be actively involved in its development. All clinicians must inform their patients the predicted number of sessions, what kind of intervention it would be, what are the key indicators of remission, and a discharge process at the end. Forming a therapeutic alliance with patients is a key determinant of treatment efficacy for psychiatric disorders. While building trust is a challenging exercise, once the therapeutic alliance is established, treatment adherence could significantly improve.

This trial also provides potential learning points for other countries, which may not be considered Low or Middle-Income, but have similarly limited resources for mental health services due to an imbalance of supply and demand. Stigma towards mental health issues, and by proxy, stigma towards consultations with mental health professionals, is not limited to LMICs. Migrant populations might carry the stigma with them. In an increasingly globalised world, societies are becoming multi-cultural, and as a result, mental health systems must be prepared to catch those who would otherwise fall through the cracks. In contexts where the waiting time to see a specialist could result in further detriment to the patient, providing mental health services for mild to moderate conditions within primary care is a clinically effective temporary solution, until the number of specialists reach acceptable levels.

While there are clear arguments for the integration of mental health into primary care, Saraceno et al. (2007) noted the three key barriers to the integration of mental health into primary care. Firstly, the overburdened primary care means that GPs may not have the time to provide adequate mental health care. Secondly, GPs do not receive regular supervision and support for mental health care, which may result in inappropriate treatment. Lastly, essential psychotropic medication may not be available. It has been argued that the role of mental health specialists, including psychiatrists, psychologists, and mental health nurses, among others, should transform from service provision to training and supervision [75].

Further health services and delivery research is required to explore how best to combine available resources and package an implementable procedure. As the ultimate consumers and beneficiaries, patients’ preferences should be considered not only in the development of an effective approach but also when deciding to scale-up the programme across the country. Patient preferences are often disregarded within clinical settings [76]. Despite this, they are increasingly regarded as key to ensure the development of a mental health system that patients feel comfortable to access and do actually access. An often-neglected perspective, the people coming to the service should be consulted when planning a scale-up of existing services, as ultimately, it is they who will either benefit or not from the service.

## Supporting information

S1 Fig Participant flow diagram for economic analyses. (TIFF)

S2 Fig Cluster recruitment. (TIFF)

S1 File Trial protocol. (DOCX)

S2 File Detail on the Malaysian value set for EQ-5D-3. (DOCX)

S3 File Sample size calculation. (DOCX)

S4 File Unit costs of Puskesmas mental health services. (DOCX)

S5 File Details of cost-effectiveness analysis and cost-utility analysis. (DOCX)

S6 File CONSORT 2010 checklist. (DOC)
