# Supplementary figures and images for: Can General Practitioners manage mental disorders in primary care? A partially randomised, pragmatic, cluster trial
Source: PLoS One. 2019 Nov 7;14(11):e0224724. doi: 10.1371/journal.pone.0224724 (PMC6837310; doi:10.1371/journal.pone.0224724)

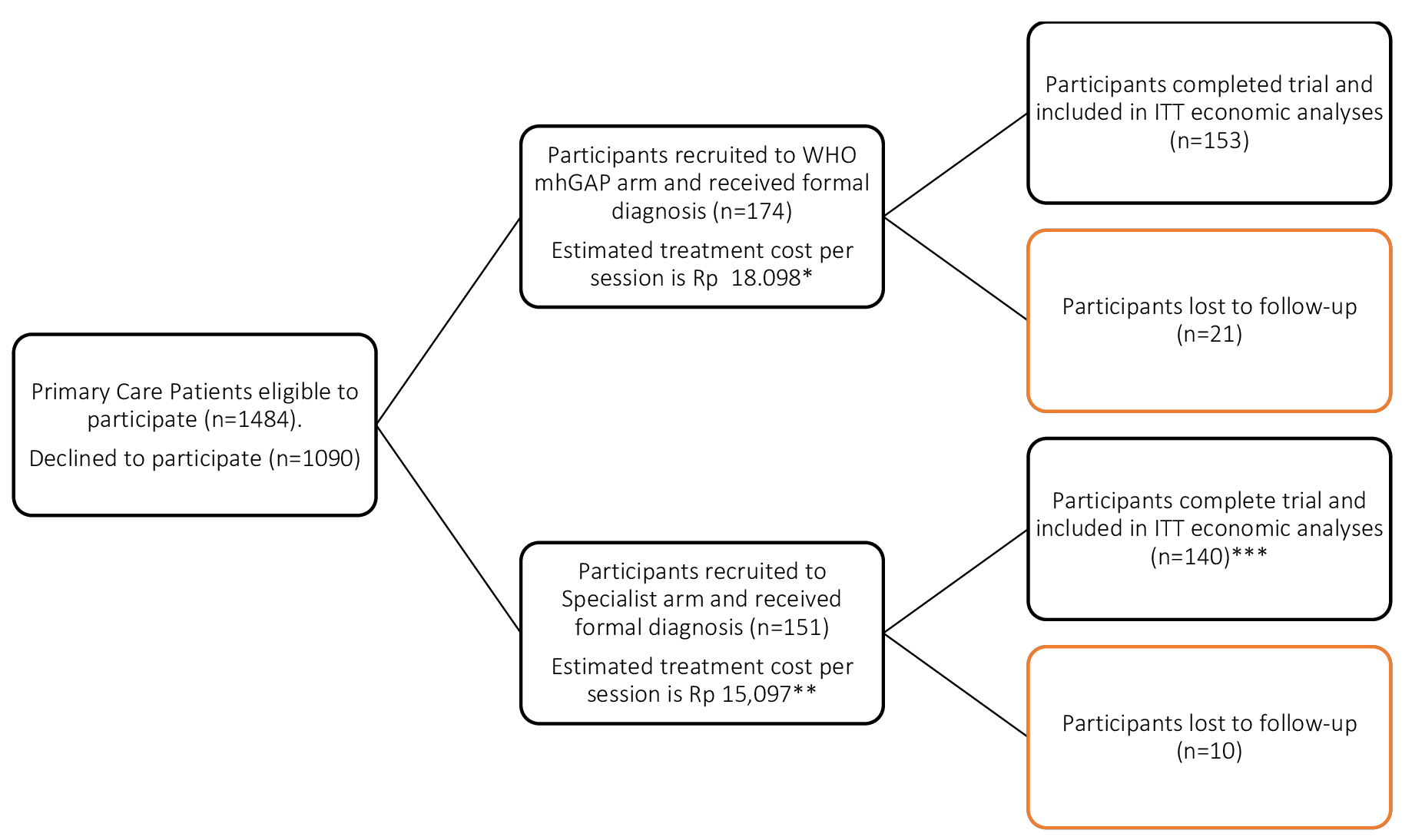

Supplement: S1 Fig — (TIFF) [file pone.0224724.s001.tiff]

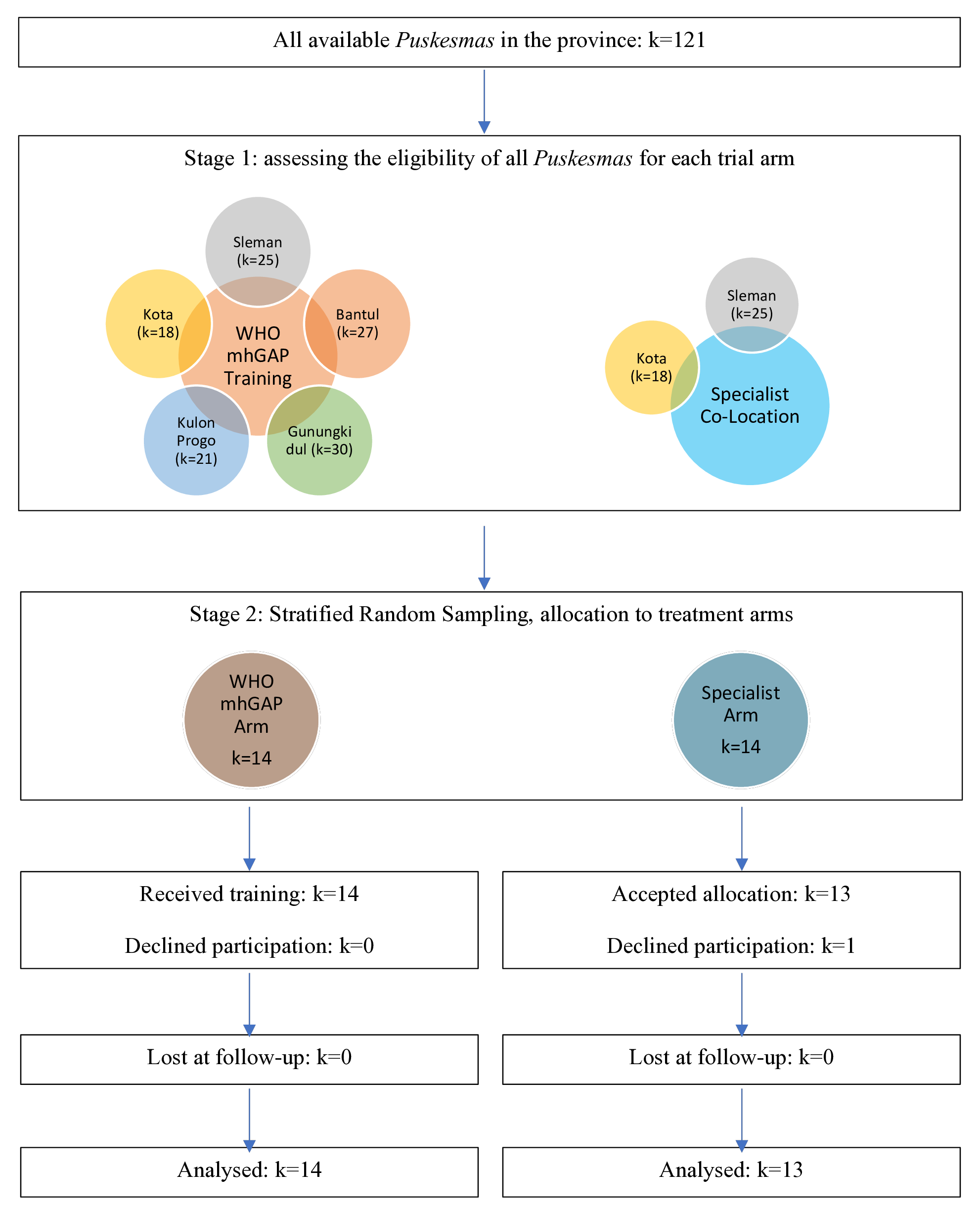

Supplement: S2 Fig — (TIFF) [file pone.0224724.s002.tiff]
