# Supplementary material for: Can General Practitioners manage mental disorders in primary care? A partially randomised, pragmatic, cluster trial
Source: PLoS One. 2019 Nov 7;14(11):e0224724. doi: 10.1371/journal.pone.0224724 (PMC6837310; doi:10.1371/journal.pone.0224724)
Supplement: S1 File — (DOCX) [file pone.0224724.s003.docx]

Trial Protocol

## Study Design

This study (“the trial”) set out to compare patient outcomes following usual care either by primary care doctors additionally trained in the Indonesian-adapted WHO mhGAP framework or clinical psychologists in the Specialist Co-location framework through a **pragmatic**, two-arm **cluster partially randomised non-inferiority trial**. This study design enabled an examination of patients derived from whole populations in a ‘real world’ setting.

A standard randomised controlled trial (RCT) would require both frameworks of services to be provided within each site, i.e. the *Puskesmas* unit. Within the context of this trial, participants randomised to another treatment group would be asked to travel to another location if the current *Puskesmas* does not provide the framework. Having both treatment frameworks within a *Puskesmas* unit was not desirable due to potential contamination between intervention and control participants. Contamination of control participants would have two effects: reducing the point estimate of an intervention’s effectiveness and, as a result, a higher likelihood of type II error. Considering potential contamination, a pragmatic CRCT design was chosen, despite requiring a bigger sample size for the same statistical power and the possibility of increased recruitment bias.

A non-inferiority trial was chosen to test whether either framework is better than the other, for all outcomes. This type of trial is frequently used where the use of a superiority trial against a placebo control (e.g. no treatment) is considered unethical.

The primary outcome was the health and social functioning of participants as measured by the Health of the Nation Outcome Scale (**HoNOS**) [1] and secondary outcomes were disability as measured by WHO Disability Assessment Schedule 2.0 (**WHODAS 2.0**) [2], quality of life as measured by European Quality of Life Scale (**EQ‐5D-3L**) [3], and **cost of intervention** evaluated from a health services perspective, which aimed to determine the clinical effectiveness and cost-effectiveness of the adapted WHO mhGAP framework of primary mental health care versus a Specialist framework of care at 6-month follow-up. Both are currently operational frameworks of primary mental health service provision in several *Puskesmas* within one province (Yogyakarta) in Indonesia. The selection of outcome measures was advised by Dr S. Idaiani from the Indonesian Ministry of Health.

The trial protocol was assessed during a University of Cambridge internal examination on 19^th^ November 2015, by Dr Stephen Gillam and Professor Martin Roland.

The trial involved two phases: a pilot study in June 2016 with the objectives to refine data collection procedures and to serve as a practice run for clinicians involved in the trial; as well as a substantive trial beginning in December 2016. This chapter describes the methods and outcomes of the pilot study, with a focus on the impact on the recruitment strategies used for the substantive trial. Subsequently, this chapter described the methods used for the substantive trial.

## Pilot Study (June 2016)

A pilot study is defined by the UK’s National Institute for Health Research (NIHR) as:

“… a version of the main study that is run in miniature to test whether the components of the main study can all work together. It is focused on the processes of the main study, for example to ensure recruitment, retention, randomization, treatment, and follow-up assessments all run smoothly.”

The pilot study, therefore, resembled the substantive study in most respects, except for follow-up assessments, given the time limitation of the doctoral degree. The recruitment period for the pilot was one week, and was designed to assess three questions:

1. Is it feasible to recruit the number of participants required to meet the planned sample size?
2. Is the screening and recruitment procedure feasible?
3. Do clinicians find it difficult to perform the in-depth psychiatric interview required to formalise a diagnosis, under time pressure?

### Results of the Pilot Study

Concerning question 1, the internal pilot proved that recruitment was feasible if conducted over a more extended period and that the planned sample size could be achieved in principle. The pilot recruitment graph is presented in *Appendix C*.

The pilot study was conducted over a period of one week in June 2016. Trained and vetted research assistants checked in for duty every morning at 7 am and were asked to take a self-portrait with a specific object within the *Puskesmas* of their duty. Information on the object, e.g. queue number collection counter, was provided every morning to ensure compliance. A tally of the number of screenings completed was checked against *Puskesmas* attendance at the end of every day, which enabled the calculation of the percentage of adult primary care attendees screened. Research assistants checked out from duty by sending a picture of the empty *Puskesmas* to the PhD Researcher coordinating the study on site. All research assistants were given the same base salary (Rp 100,000 ~ £6, per day), but those on duty at a *Puskesmas* more than three hours’ drive from the centre of Yogyakarta were given additional transport allowance of a day’s salary. Given the lack of an incentive structure, I observed a steady decrease in the percentage of adult attendees screened daily in some *Puskesmas,* indicating a decline in commitment which prompted me to consider an incentive structure for the substantive study. Nonetheless, 5341 patients were screened within the recruitment week.

The evaluation of question 2 indicated that the provision of one research assistant per clinic to manage the screening of all adult primary care attendees raised minor challenges. As the research assistant had to conduct recruitment by explaining the trial objectives to those meeting the screening criteria, several adult primary care attendees were left unscreened every day. To further complicate matters, older patients required help with reading the screening questionnaire which rendered other patients neglected. It was also tricky for research assistants to manage four separate documents for each patient: screening questionnaire, information sheet, informed consent sheet, and the questionnaire booklet. All this need to be compiled with clinician’s assessment following the psychiatric interview. I noticed the potential for the mismanagement of paperwork by research assistants. Despite the challenges, the proposed procedure proved to be feasible. A focus group with five research assistants and local adviser, Dr Bambang Hastha Yoga, discussed potential mitigating strategies for the substantive trial, including having a pair of research assistants per *Puskesmas*, which were eventually implemented in the substantive trial.

The evaluation of question 3 indicated an inconsistency of confidence among clinicians to conduct in-depth psychiatric interviews during busy clinic hours. The pilot study highlighted that with limited time, such as during general practitioner’s consultation, it was difficult for clinicians to ask questions relating to symptoms of psychiatric morbidity. I was interested in also conducting a Receiver Operating Curve study on the GHQ-12 a gold standard against which the threshold scores could be compared to was required. Given the precedent set by similar studies conducted in sub-Saharan Africa, under the advice of Professor Martin Prince of King’s College London, I incorporated the Revised Clinical Interview Schedule (CIS-R) which is a structured interview guide to assess the symptoms of psychiatric disorders efficiently.

Subtle changes to the substantive study were made, including assigning two research assistants per clinic and converting the information sheets, informed consent sheets, questionnaire batteries, and structured psychiatric interview guide into booklet form. In addition, I extended the recruitment period of the substantive study to two weeks. Doubling the number of research assistants and the length of the recruitment period raised logistical and resource challenges, as additional recruitment and training were required in addition to extra research expenses.

## Main Trial Methods

### Ethics and governance

Ethics approval for the study was granted by the University of Cambridge Psychology Research Ethics Committee (reference number PRE.2015.108) and Universitas Gadjah Mada (reference number 1237/SD/PL.03.07/IV/2016). Trial insurance further covers investigators and research participants (University of Cambridge Trial Insurance reference number 609/M/C/1510). Ethics approval from all the clusters was not required as each cluster (*Puskesmas*) is a state-owned clinic funded and managed by district governments. Permission to conduct research at the Province of Yogyakarta including its all five districts was obtained from the Provincial Government Office (reference number 070/REG/V/625/5/2016). This trial is registered with clinicaltrials.gov on 25 February 2016, NCT02700490.

### Interventions

#### Experimental Group: Indonesian-adapted WHO mhGAP

The concept of “Treatment Gap” (TG -- % of those unidentified and untreated who might benefit from known treatments) has been identified as a critical issue within global mental health, leading to the WHO response that is the Mental Health Gap Action Programme (WHO mhGAP). The training manual is available for free download online in various languages, at <http://www.who.int/mental_health/mhgap/training_manuals/en/>. Since 2008, the WHO mhGAP programme aims to scale up services for mental, neurological, and substance use disorders especially among low and middle-income countries, by providing add-on clinical mental health training to a non-specialist audience. The intervention guide, first released in 2010, presents integrated management of priority conditions including depression, psychosis, bipolar disorder, epilepsy, developmental and behavioural disorders in children and adolescents, dementia, disorders due to substance abuse, self-harm/suicide and other significant emotional or medically unexplained complaints.

Proposed management of disorders includes psychoeducation, basic and advanced psychosocial interventions, and pharmacological therapy. Advanced psychosocial interventions outlined in the WHO mhGAP Intervention Guide 1.0 are listed in the WHO website ([https://www.paho.org/mhgap/en/int_management.html – accessed on 26 October 2018)](https://www.paho.org/mhgap/en/int_management.html%20–%20accessed%20on%2026%20October%202018)) and include: Behavioural Activation, Cognitive Behavioural Therapy, Contingency Management Therapy, Family Counselling or Therapy, Interpersonal Psychotherapy, Problem-solving Therapy, Relaxation Training, and Social Skills Therapy.

The WHO mhGAP framework has been translated to *Bahasa Indonesia* by the Indonesian Ministry of Health in 2015, adapted to the country’s context, and systematically introduced throughout the network of 10,000 *Puskesmas* across the country. The implementation of the adapted WHO mhGAP framework aims to reduce Treatment Gap through facilitating early identification and providing basic or initial intervention in primary care. Since its adaptation and adoption in late 2015, Indonesia’s Directorate of Mental Health trained between 10 to 20 pairs of *Puskesmas* doctors (general practitioners) and *Puskesmas* nurses from each province every month, alternating between 34 provinces. These *Puskesmas* were chosen at random from all available *Puskesmas*, with the intention of scaling up to all 10,000 *Puskesmas* across the country.

The Ministry of Health designated the adapted WHO mhGAP framework as a scale-up training for registered medical practitioners and nurses who already had to complete a set of competencies for a comprehensive list of psychiatric conditions prior to gaining a license to practice. The adapted WHO mhGAP modules are therefore, in the Indonesian context, akin to the addition of new tools to an already comprehensive toolbox of a medical doctor. It should be noted that the set of competencies and psychiatric conditions agreed the Indonesian Medical Council and listed in the Indonesian Medical Practitioners Competency Standards are specific to the Indonesian context, (<http://fk.ub.ac.id/profesi/wp-content/uploads/2013/10/Standar-Kompetensi-Dokter-Indonesia_SKDI-2012-1.pdf>) in light of the limited provision of specialist medical care in rural Indonesia, and may not be transferable to other countries. WHO mhGAP practitioners typically employ a combination of basic psychosocial intervention and pharmacological therapy to manage disorders.

For the list of Medical Doctor Basic Licensing core competencies in Table 1, there are four levels of competency determined:

1. Level 1: theoretical understanding including biomedical and psychosocial aspects, as well as the ability to explain to patients and family members, colleagues, and other professions including regarding the principles, indications, and complications which may arise.
2. Level 2: theoretical and practical understanding, including biomedical and psychosocial aspects, with emphases on clinical reasoning and problem solving, including direct observation experience of patients.
3. Level 3: theoretical and practical understanding, including biomedical and psychosocial aspects, with direct practical experience on standardised patients (e.g. through Objective Structured Clinical Examinations (OSCE)).
4. Level 4: independent practice, following thorough theoretical and practical understanding and experience, practice under direct supervision, for example through work-based assessments (e.g. through mini-CEX).
5. 4A: core competencies derived upon completing general medical education.
6. 4B: proficiencies derived after internship.

Table 1. Medical Doctor Basic Licensing Core Competencies for Psychiatry, Medical Practitioners Competency Standards (source: Indonesian Medical Council)

| **Number** | **Competency** | **Level of Competency** |
| --- | --- | --- |
| ANAMNESIS | | |
|  | Autoanamnesis with patient | 4A |
|  | Alloanamnesis with other family members or significant others | 4A |
|  | Information on primary presenting problem | 4A |
|  | History of the progress of condition | 4A |
|  | Gaining meaningful information regarding early childhood development, education, employment, relationship, family life | 4A |
| PSYCHIATRIC ASSESSMENT | | |
|  | Assessment of mental state | 4A |
|  | Assessment of consciousness | 4A |
|  | Clinical assessment of perception, orientation, and intelligence | 4A |
|  | Assessment of orientation | 4A |
|  | Clinical assessment of intelligence | 4A |
|  | Assessment of form and content of thought | 4A |
|  | Assessment of mood and affect | 4A |
|  | Assessment of motor function | 4A |
|  | Assessment of impulse control | 4A |
|  | Assessment of judgment ability | 4A |
|  | Assessment of insight | 4A |
|  | Assessment of general functioning | 4A |
|  | Personality assessment (projective, inventory, etc.) | 2 |
| DIAGNOSIS AND IDENTIFICATION | | |
|  | Establishing a diagnosis based on multiaxial diagnostic criteria | 4A |
|  | Differential diagnosis | 4A |
|  | Identifying psychiatric emergency | 4A |
|  | Identifying physical, psychological, and social problems | 4A |
|  | Considering prognosis | 4A |
|  | Determining reason for referral | 4A |
| ADDITIONAL ASSESSMENT | | |
|  | Mini Mental State Examination | 4A |
|  | Home visit, if required | 4A |
|  | Consultative cooperation in a multidisciplinary team | 4A |
| THERAPY | | |
|  | Psychopharmacology therapy (antipsychotic, antidepressant, anticholinergic, sedative, etc.) | 3 |
|  | Electroconvulsive therapy (ECT) | 2 |
|  | Supportive psychotherapy (counselling) | 3 |
|  | Behaviour modification therapy | 2 |
|  | Cognitive Behaviour Therapy (CBT) | 2 |
|  | Psychoanalytic psychotherapy | 1 |
|  | Hypnotherapy and Relaxation therapy | 2 |
|  | Group Therapy | 1 |
|  | Family Therapy | 2 |

For the list of psychiatric conditions which are covered in the Medical Practitioners Competency Standard (Table 2), there are also four levels of competencies expected:

1. Level 1: understanding and explaining the clinical presentation of the condition and able to utilise the most suitable method to gain further information on the condition, afterwards determining the appropriate referral pathway for the patient. Graduates of medical school are also able to manage the condition following return from referral.
2. Level 2: able to form a clinical diagnosis of the condition, and determining the appropriate referral pathway for follow-up intervention. Graduates of medical school are also able to manage the condition following return from referral.
3. Level 3: diagnosing, providing initial intervention, and referring.
4. Level 3A: in non-emergency situations, graduates of medical school are able to form a clinical diagnosis and provide initial intervention. Medical school graduates are then able to determine the appropriate referral pathway for follow-up intervention. Graduates of medical school are also able to manage the condition following return from referral.
5. Level 3B: in emergency situations, graduates of medical school are able to form a clinical diagnosis and provide initial intervention to save patient’s life or prevent further harm on the patient. Medical school graduates are then able to determine the appropriate referral pathway for follow-up intervention. Graduates of medical school are also able to manage the condition following return from referral.
6. Level 4: diagnosing, managing condition independently, and discharging.
7. Level 4A: core competencies derived upon graduation from medical school.
8. Level 4B: proficiencies derived after internship.

Table 2. ICD-10 Psychiatric Conditions which are part of Medical Doctors' Basic Licensing Core Competencies (source: Indonesian Medical Council)

| **Number** | **Conditions** | **Level of Competency** |
| --- | --- | --- |
| ORGANIC MENTAL DISORDER | | |
|  | Delirium not induced by alcohol or other psychoactive substances | 3A |
| MENTAL AND BEHAVIOURAL DISORDERS FROM SUBSTANCE USE | | |
|  | Acute intoxication of psychoactive substance | 3B |
|  | Addiction/substance dependency | 3A |
|  | Delirium induced by alcohol or other psychoactive substances | 3A |
| PSYCHOSIS | | |
|  | Schizophrenia | 3A |
|  | Delusion | 3A |
|  | Psychotic disorder | 3A |
|  | Schizoaffective disorder | 3A |
|  | Bipolar disorder, manic episode | 3A |
|  | Bipolar disorder, depressive episode | 3A |
| NEUROTIC DISORDERS, STRESS, AND SOMATOFORM DISORDERS | | |
|  | Cyclothymic disorder | 2 |
|  | Endogenic depression (single episode or recurrent) | 2 |
|  | Dysthymic disorder (neurosis depression) | 2 |
|  | Depressive episode (unclassified) | 2 |
|  | Baby blues (post-natal depression) | 2 |
| ANXIETY AND PHOBIA | | |
|  | Agoraphobia with or without panic | 2 |
|  | Social phobia | 2 |
|  | Specific phobia | 2 |
| OTHER ANXIETY DISORDERS | | |
|  | Panic disorder | 3A |
|  | General anxiety disorder | 3A |
|  | Mixed anxiety and depression | 3A |
|  | Obsessive-compulsive disorder | 2 |
|  | Reaction towards stress and adjustment disorder | 2 |
|  | Post-traumatic stress disorder | 3A |
|  | Dissociative disorder (conversion) | 2 |
|  | Somatoform disorder | 4A |
|  | Trichotillomania | 3A |
| PERSONALITY DISORDER AND ADULT BEHAVIOURAL DISORDERS | | |
|  | Personality disorder | 2 |
|  | Gender identity disorder | 2 |
|  | Sexual preference disorder | 2 |
| EMOTIONAL AND BEHAVIOURAL DISORDERS, CHILDHOOD ONSET | | |
|  | Pervasive developmental disorder | 2 |
|  | Mental retardation | 3A |
|  | Attention deficit disorder, hyperactive disorder (including autism) | 2 |
|  | Conduct disorder | 2 |
| EATING DISORDERS | | |
|  | Anorexia nervosa | 2 |
|  | Bulimia | 2 |
|  | Pica | 2 |
| TICS | | |
|  | Tourette’s syndrome | 2 |
|  | Chronic motor or vocal tics disorder | 2 |
|  | Transient tics disorder | 3A |
| EXCRETORY DISORDERS | | |
|  | Functional encoperasis | 2 |
|  | Functional enuresis | 2 |
| SPEECH DISORDER | | |
|  | Uncoordinated speech | 2 |
| SEXUAL DYSFUNCTION DISORDER | | |
|  | Paraphilia | 2 |
|  | Sexual desire or arousal disorder | 3A |
|  | Female orgasmic disorder or premature ejaculation | 3A |
|  | Sexual pain disorder (including vaginismus, dyspareunia) | 3A |
| SLEEP DISORDERS | | |
|  | Insomnia | 4A |
|  | Hypersomnia | 3A |
|  | Sleep-wake cycle disturbance | 2 |
|  | Nightmares | 2 |
|  | Sleep walking | 2 |

In the adapted WHO mhGAP Arm, *Puskesmas* doctors provided pharmacological therapy and/or psychosocial intervention, and/or referred participants to specialist care, as they saw fit. As the trial was designed to reflect real-life practice, *Puskesmas* doctors’ choice of intervention was not recorded, nor their utility of the adapted WHO mhGAP modules enforced.

#### Experimental Group: Specialist Co-location

The Specialist framework is the integration of clinical psychologists into the primary care system. It is considered an important step towards scaling up mental health services [4]. There is good evidence for the effectiveness of psychotherapy delivered in primary care [4] and that psychotherapy is as effective as antidepressant medication [5]. The co-location of clinical psychologists in primary care improves collaboration and potentially reduce the stigma of mental illness and barriers to care [6-8].

In 2004, the Sleman District Health Office (within Yogyakarta province), in collaboration with the Centre for Public Mental Health (Universitas Gadjah Mada), initiated the integration of clinical psychologists within primary care [9]. Following a Bachelor’s Degree in Psychology and a Master’s Degree in Clinical Psychology, one is required to obtain professional registration as a Clinical Psychologist before seeking employment at a *Puskesmas*. In 2016, psychologists were employed in 43 of 121 *Puskesmas* in Yogyakarta province, specifically in all *Puskesmas* within the districts of Sleman and Kota. In the Specialist framework, clinical psychologists typically use a combination of basic and advanced psychosocial intervention to manage disorders. As the trial was designed to reflect real-life practice, *Puskesmas* psychologists’ choice of intervention for research participants was not recorded.

### Clinician training

Indonesia's Directorate of Mental Health provided health care providers training in the Indonesian-adapted WHO mhGAP framework. In April 2016 the week-long training of *Puskesmas* General Practitioners took place in Yogyakarta, the province where the trial took place. A psychiatrist from Indonesia’s Directorate of Mental Health who had gone through a WHO mhGAP train-the-trainer course conducted the general practitioners’ training. The week-long training covered all WHO mhGAP modules, and further incorporated a day of role-playing plus clinical observations in ‘real life’ setting.

In the Specialist framework, Clinical Psychologists received their mental health assessment and therapy training during their two-year Master’s Degree in Clinical Psychology. All psychologists involved in the trial had professional registration as a psychologist in Indonesia. *Puskesmas* psychologists attend regular continuing professional development (CPD) training organised by the Centre for Public Mental Health, a collaborator of this trial.

Before the commencement of the Pilot Study, all clinicians involved in the trial attended a one-day training session on the questionnaires used and on in-depth psychiatric interviews in non-specialised health settings. I conducted the training on the use of questionnaires (WHODAS 2.0, EQ-5D), while a renowned local psychiatrist conducted the training on psychiatric interviewing to complete the primary outcome measure, HoNOS. The training focused on the standardisation of scoring on primary and secondary outcome measures.

Before the commencement of the Substantive Study, all clinicians involved in the trial attended a one-day training session on structured psychiatric interviews in non-specialised health settings (CIS-R). As a psychologist trained in Australia and the UK, with several years of clinical experience, I conducted the training on the administration of the questions and classification of responses, going through each item and facilitating role-play in pairs. Clinicians were also introduced to the research assistants who would be based at their clinics. Clinicians were not trained to score or interpret the result of the CIS-R, as the utility of the CIS-R in the trial was limited to the standardised way of asking clinical questions, analysis of the GHQ-12 as an effective screening tool, and post-hoc comparisons at the end of the trial period. Clinicians did not use the CIS-R score to establish a diagnosis.

### Adherence and fidelity

As this is a pragmatic trial conducted in ‘real-life’ setting, clinicians were informed that only adherence to the interview protocol (baseline and a follow-up at 6-months) would be enforced, while the onus was on them to ensure service users’ treatment adherence, as per usual practice. To facilitate buy-in and avoid the Hawthorne Effect, the trial did not capture any record of clinicians’ choice of intervention. The trial did not evaluate the accuracy of diagnosis, nor the appropriateness of the chosen intervention for the diagnosis given. As such, clinicians had the freedom to choose from among their repertoire of interventions, which they developed either through their adapted WHO mhGAP training, clinical psychology training, or any prior training and development activities, without feeling monitored.

Fidelity to chosen treatment procedures was not enforced as this study hoped to reflect actual practices on the ground, which may be adjusted by clinicians based on service users’ responsiveness to treatment. As such, the intention-to-treat (ITT) approach is applied to the design, conduct, and statistical analysis of the trial. The ITT approach avoids overoptimistic estimates of the efficacy and feasibility of an intervention resulting from the removal of non-compliers from trials or data analysis [10].

### Setting

Yogyakarta is a province in Java, the only place in Indonesia where the specialist co-location framework is operational at the start of the trial. It is crucial for any ‘real life’ evaluation to take place in the original setting and for any comparison framework to be introduced to a context as similar as possible to the original [11]. There are five districts in Yogyakarta Province: Kota, Sleman, Bantul, Kulon Progo, Gunungkidul, and among them 121 *Puskesmas*. Sleman district in Yogyakarta province also pioneered the specialist primary mental health service model in 2004. Before the start of the trial, the Specialist framework was present in only 43 *Puskesmas* in Kota and Sleman districts.

The adapted WHO mhGAP has been systematically introduced to *Puskesmas* within Indonesia since 2015 but was not yet operational in most *Puskesmas*. The adapted WHO mhGAP framework was introduced to *Puskesmas* in Yogyakarta in an experimental manner (randomised), enabling a pragmatic cluster randomised trial to take place.

The trial took place in primary care clinics (*Puskesmas*) in Yogyakarta province. Stratified random sampling was considered the optimum allocation for the estimation of population means when considering a multivariate problem. The *Puskesmas* were assigned to each treatment arm in a 1:1 ratio. The number of *Puskesmas* per district invited to participate in the study was in proportion to each district’s population size.

All *Puskesmas* in Yogyakarta had received International Organisation for Standardization (ISO) Certification, so that data collection points within routine patient flow procedures could be embedded.

### Randomisation and blinding

Clusters (*Puskesmas*) were randomised (in a ratio of 1:1) to either treatment arm. Randomisation was done by our External Statistical Adviser, Dr Chiara Bonetto, using stratified random sampling. Within the study design, the unit of randomisation was the *Puskesmas,* and the unit of observation and analysis was the service user. The cluster model allowed service providers to adhere as a ‘whole service’ to the treatment method they were trained to provide.

The clustered nature of the study allowed patient-participants to be blinded to the existence of an alternative treatment pathway. Outcome assessments were conducted during home visits, and by a different assessor (trained research assistant) blinded to treatment allocation.

### Site recruitment

Training dates, logistics, and written support from the Ministry of Health, Provincial Health Authority, and District Health Authorities facilitated the recruitment of all 14 sites to the trial. For the Specialist arm, 14 randomly chosen *Puskesmas* with existing clinical psychologist co-location were recruited. Letters of approval to conduct research by the Ministry of Health and the Provincial Health Authority were sent to all 28 *Puskesmas*, along with an information pack about the trial. A one-day briefing was held with Heads of *Puskesmas*, all clinicians involved, and representatives of District Health Authority, hosted and organised by the Provincial Health Authority. Each *Puskesmas* was assigned a unique cluster number.

All clinicians were current treatment providers in *Puskesmas* and were employees of the District Health Authorities. Therefore, they were not hired specifically for the study, rather the study was integrated into their work. Following a discussion with the Head of Medical Services of the Province in 2016, to ensure that screening and treatment procedures introduced during the trial continue to be integrated as standard operating procedures in each *Puskesmas*, clinicians were not paid to participate in the trial.

It was anticipated that clinicians would fear being pitted against each other, as the trial sought to compare clinical and cost-effectiveness. Three strategies were used to foster cooperation. Firstly, clinicians were introduced to the idea of non-inferiority trial. Secondly, rather than using primary measures such as diagnostic accuracy, discharge rates, and length of treatment, ‘proxy measures’ such as symptom reduction at six months, as well as overall health and social functioning (primary outcome measure) reduced the spotlight on individual clinicians. Thirdly, conducting joint training sessions where clinicians got to know each other and ate together, fostered a collegiate atmosphere and the idea that everyone works towards a common goal.

### Patient recruitment

Participants were adult primary care attendees visiting any of the 28 *Puskesmas* during the recruitment period, and not currently on any psychosocial or pharmacological therapy for mental health issues, who met the screening criteria (GHQ-12 score of 2 or above, GHQ scoring method 0-0-1-1) [12]. Primary care attendees who self-referred to the psychology service were invited to participate if they did not have ongoing treatment for mental health issues. Those receiving ongoing treatment for mental health issues were excluded from participating in the trial.

During the recruitment period, all adult primary care patients in participating clusters (estimated at 40 patients per day) were given the screening questionnaire, the General Health Questionnaire (GHQ-12) [12], as well as a demographic questionnaire from the Client Service Receipt Inventory (CSRI) by Beecham and Knapp, 2001). The CSRI is a generic questionnaire that records demographics information and health services utility within a specific period (e.g. six months) [13]. Participants were given the screening questionnaire at the registration counter, when they obtained a queue number, to be self-completed while waiting for routine blood pressure test.

Primary care patients were given the screening questionnaire at the registration counter, when they obtained a queue number, to be self-completed while waiting for routine blood pressure test. Research assistants trained in scoring the GHQ identified patients who met the screening criteria. To ensure avoidance of any sense of coercion, participants were asked to provide written informed consent before meeting a clinician and were also reassured that declining would not affect usual medical care. Participants were invited to ask questions for clarification. Research assistants double-checked potential participants’ understanding of the follow-up requirements and their rights to withdraw. Research assistants provided a brief overview of the trial and an information sheet. Full consent with signature was requested from those who agreed to take part. Participants were then given a questionnaire booklet to complete while waiting to meet either the *Puskesmas* doctor or psychologist, depending on which cluster they were at.. The questionnaire booklet contained the self-complete version of the WHO Disability Assessment Schedule 2.0 (WHODAS 2.0) [2] and the European Quality of Life Scale (EQ‐5D-3L) [3]. Participants were also informed that they were free to withdraw at any time during the study.

There were procedures in place to assist illiterate patients, which were not required during the recruitment period of this trial. For these patients, consent form would have required the participant’s thumbprint and the signature of a witness. Illiterate patients would have been described the research aims and procedures as usual, but rather than self-completing the questionnaire booklet, a research assistant would interview them, completing the booklet alongside.

In the WHO mhGAP treatment arm, participants’ medical records and questionnaire booklets were passed to the ***Puskesmas* doctor** who had received the adapted WHO mhGAP training from the Ministry of Health. Standard medical consultations took place, followed by a structured interview comprising the CIS-R and HoNOS, located in the second half of the questionnaire booklet (Appendix I). *Puskesmas* doctors would then record participants’ names, the medical record number, and contact phone number or home address separately. Plans for medications and/or psychosocial therapy, if deemed necessary, were developed together with participants and they were asked to return for further therapy sessions.

In the Specialist arm, participants’ medical records were passed to a general practitioner along with a request for a referral to a psychologist. Standard medical consultations for any physical ailments took place with general practitioners before participants went to a psychology consultation room for a structured interview with the ***Puskesmas* psychologist**. The psychologist then completed both the CIS-R and HoNOS, located in the second half of the questionnaire booklet. Psychologists would then record separately participants’ names, the medical record number, and contact phone number or home address. If therapy was deemed necessary, participants were asked to return for further therapy sessions.

The questionnaire booklet incorporated a section where the clinician could indicate participant’s diagnosis at the end of the in-depth interview. This diagnosis was not determined by the CIS-R score, but rather was based on the clinical judgment of each Puskesmas doctor or clinical psychologist. This diagnosis determines whether participants were asked to return for intervention sessions.

### Follow-up assessment

Trained and vetted research assistants blinded to treatment arm allocation conducted follow-up at six months. They obtained participants’ contact details from their *Puskesmas* and contacted participants via telephone from the *Puskesmas* approximately a month to a fortnight before follow-up home visit. Participant personal details including phone numbers were not shared with other members of the research team and were kept confidential by each field researcher. Participants were re-assessed using the full battery of instruments. At this point, no additional/replacement diagnosis was assigned to the participants.

Prior to the home-visits taking place, all field researchers met the PhD researcher to go through mitigation strategies in the event of a crisis (e.g. if a participant becomes distressed or threatening). Follow-up interviews were conducted without any other persons present. To ensure safety for both the researchers and participants, the room must be unlocked. Field researcher’s vehicle (moped) was required to remain parked outside the property of the participant, and the personal properties of field researchers must remain with them at all times. They were required to have a mobile phone, fully charged, and able to make emergency phone calls if required during the home visit. A representative from the *Puskesmas* must accompany field researchers during the home visit.

Participants were re-assessed using the full battery of instruments: WHODAS 2.0 (self-report), EQ-5D (self-report), CIS-R (during the clinical interview), and HoNOS (post-clinical interview). At this point, no additional/replacement diagnosis was assigned to the participants.

The schedule of enrolment, interventions, and assessments is shown in Table 3, based on the Standard Protocol Items: Recommendations for Interventional Trials (SPIRIT) [14].

Table 3. Modified SPIRIT Checklist

|  | STUDY PERIOD | | | | | |
| --- | --- | --- | --- | --- | --- | --- |
|  | Allocation | Enrolment | Post-enrolment | | | Close-out |
| TIMEPOINT** | -t_1_ | 0 | t_1_ | t_2_ | etc. | t_x_ |
| RANDOMISATION  of Clusters | X |  |  |  |  |  |
| ENROLMENT: |  |  |  |  |  |  |
| Eligibility screen |  | X |  |  |  |  |
| Informed consent |  | X |  |  |  |  |
| Baseline Interview |  | X |  |  |  |  |
| Diagnosis |  | X |  |  |  |  |
| INTERVENTIONS: |  |  |  |  |  |  |
| WHO mhGAP |  |  |  |  |  |  |
| Clinical Psychology |  |  |  |  |  |  |
| ASSESSMENTS: |  |  |  |  |  |  |
| CIS-R |  | X |  |  |  | X |
| HoNOS |  | X |  |  |  | X |
| WHO mhGAP |  | X |  |  |  | X |
| EQ-5D-3L |  | X |  |  |  | X |
| Health Services Use |  | X |  |  |  | X |

### Measures

#### Screening

The twelve-item General Health Questionnaire (GHQ-12) was developed to screen for general (non-psychotic) psychological morbidity among primary care patients (Goldberg & Williams, 1988). Items on the GHQ-12 are rated on a 4-point scale using a time-frame of ‘in the last two weeks’. There are two ways of scoring the GHQ-12: the bimodal GHQ scoring method (0-0-1-1) recommended by the test authors for use in clinical settings; and the Likert scoring method (0-1-2-3) which is commonly used in research settings. As this ‘real life’ study took place in a clinical setting, the bimodal scoring method was used, and patients with score 2 and above were invited to participate in the study.

The screening questionnaire incorporates questions on demographic information such as gender, education, employment, living arrangement, and chronic illnesses based on the Client Service Receipt Inventory (CSRI). The CSRI by Beecham and Knapp (2001) is a generic questionnaire that records demographics information and health services utility within a specific period (e.g. 6 months). Data collected from the CSRI indicated the number of outpatient visits to *Puskesmas*, inpatient stay, and visits to the emergency [13]. Depression and anxiety have been found to increase the risk for hypertension [15, 16]. There is also a strong evidence that depression is a risk factor for diabetes mellitus [17]. The screening questionnaire is in Appendix H.

#### Primary Outcome Measure

The Health of the Nation Outcome Scale (HoNOS) [1] is a 12-item scale to rate mental health service users in various aspects of mental and social health, each on a scale of 0-4. The items are: Overactive, aggressive, disruptive or agitated behaviour; Non-accidental self-injury; Problem drinking or drug-taking; Cognitive problems; Physical illness or disability problems; Problems associated with hallucinations and delusions; Problems with depressed mood; Other mental and behavioural problems; Problems with relationships; Problems with activities of daily living; Problems with living conditions; and Problems with occupation and activities. The ratings were made once all the information became available through clinical interview and took less than 5 minutes for the clinician to complete. HoNOS Total Score is a sum of individual item scores, with higher scores indicating greater mental or social health problems.

The primary outcomes were changes from baseline to the 6-month follow-up assessment in the health and social functioning of participants, as measured by the Health of the Nation Outcome Scale (HoNOS) [1].

#### Secondary Outcome Measures

WHO Disability Assessment Schedule (WHODAS 2.0) is a generic assessment instrument for health and disability used across all diseases, including mental, neurological, and addictive disorders. The WHODAS 2.0 covers six domains of functioning, including Cognition; Mobility; Self-care; Getting along; Life activities; and Participation. This trial used the 36-item, self-administered version, taking approximately 20 minutes for self-completion. This trial used the complex scoring method based on item response theory, as advised by the WHO. It took the coding for each item separately and then used an algorithm to determine the Total score by differentially weighting the items and the levels of severity. Total scores are in a metric ranging from 0 (no disability) to 100 (full disability).

The 3-level European Quality of Life Scale (EQ-5D-3L) is a standardised self-report measure of health-related quality of life which takes approximately 5 minutes to complete. It comprises five dimensions: mobility, self-care, usual activities, pain/discomfort, and anxiety/depression, with each dimension recording responses at one of three levels: no problems, some problems, and extreme problems [18]. EQ-5D-3L ratings are commonly converted into composite utility scores using country-specific value sets, which measure people’s preferences in relation to health and weight each of the levels in each EQ-5D-3L dimension accordingly. The single utility scores represent the state of health-related quality of life for a person at any given time. While the Indonesian value set for the 5-level version of the measure (EQ-5D-5L) has been published in 2016, there is no existing value set for EQ-5D-3L.

Existing Thai and Malaysian value sets were found to be the closest alternative. The Thai value set uses the Time Trade-Off (TTO) method to estimate its valuation [19], and the Malaysian value set combines both the TTO and Visual Analogue Scale using linear additive regression [20]. Both value sets include the N3 model from the original UK Measure and Valuation in Health study. The N3 model adds an interaction variable to capture the effect of any dimension with severe health state.

The Thai value set for the EQ-5D-3L is:

Thai score = 1 - 0.202 - (0.121*mo) - (0.121*sc) - (0.059*ua) - (0.072*pd) - (0.032*ad) - (0.190*m2) - (0.065*p2) - (0.046 * a2) - (0.139 * N3),

where **mo** is mobility, **sc** is self-care, **ua** is usual activities, **pd** is pain and discomfort, and **ad** is anxiety and depression. Variable **mo** is 1 if mobility is level 2, 2 if mobility is level 3, and 0 otherwise; variable **sc** is 1 if self-care is level 2, 2 if self-care is level 3, 0 otherwise; **ua** is 1 if usual activities is level 2, 2 if usual activities is level 3, 0 otherwise; **pd** is 1 if pain and discomfort is 2, 2 if pain and discomfort is 3, 0 otherwise; **ad** is 1 if anxiety and depression is 2, 2 if anxiety and depression is 3, 0 otherwise. Variable **m2** is 1 if mobility is level 3 and 0 otherwise; **p2** is 1 if pain and discomfort is level 3 and 0 otherwise; **a2** is 1 if anxiety and depression is level 3 and 0 otherwise and; **N3** is 1 if any dimension is level 3 and 0 otherwise.

Where the Malaysian Value set for the EQ=5D-3L is:

Malaysian score = 1 – (0.067*N2) – (0.116*N3) – (0.084*m1) – (0.191*m2) – (0.097*sc1) – (0.16*sc2) – (0.053*ua1) – (0.122*ua2) – (0.054*pd1) – (0,127*pd2) – (0.081*ad1) – (0.086*ad2)

where **mo** is mobility, **sc** is self-care, **ua** is usual activities, **pd** is pain and discomfort, and **ad** is anxiety and depression. Variable **m1** is 1 if mobility is level 2, and 0 otherwise; **m2** is 1 if mobility is level 3, and 0 otherwise; **sc1** is 1 if self-care is level 2, 0 otherwise; **sc2** is 1 if self-care is level 3, 0 otherwise; **ua1** is 1 if usual activities is level 2, 0 otherwise; **ua2** is 1 if usual activities is level 3, 0 otherwise; **pd1** is 1 if pain and discomfort is 2, 0 otherwise; **pd2** is 1 if pain and discomfort is 3, 0 otherwise; **ad1** is 1 if anxiety and depression is 2, 0 otherwise; **ad2** is 1 if anxiety and depression is 3, 0 otherwise. Variable **N2** is 1 if any dimension is level 2 and 0 otherwise and; **N3** is 1 if any dimension is level 3 and 0 otherwise.

Converting EQ-5D-3L scores to both Thai and Malaysian economic values enabled outcome assessors to perform some quick comparisons for consistency of reporting. In the economic analyses of QALY, it was determined that the Malaysian value sets would be more appropriate for the Indonesian population, given the shared culture, predominant religion, and language of both countries.

For the evaluation of psychiatric morbidity, the clinician conducted a structured interview using the Revised Clinical Interview Schedule (CIS-R) [18]. The CIS-R is a fully structured diagnostic instrument that was developed from an existing instrument, the Clinical Interview Schedule (CIS), initially designed for the use of clinically experienced interviewers. The CIS was revised and developed into a fully structured interview to increase standardisation and to make it suitable to be used by trained lay interviewers in assessing minor psychiatric morbidity in the community, general hospital, occupational and primary care research. As the CIS-R specifically diagnoses mood and anxiety disorders, participants with an indication of other disorders (psychosis, sleep disorders, dementia) were asked additional questions which enabled the interviewers (clinicians) to establish an ICD-10 diagnosis.

For this trial, structured interview using the CIS-R was conducted by general practitioners or clinical psychologists in all participating *Puskesmas*. ICD-10 is widely used in the Indonesian mental health services as the national guideline for the diagnosis of psychiatric disorders (Pedoman Panduan Diagnosa Gangguan Jiwa) is the translated version of ICD-10.

Clinician’s own diagnosis of each patient was recorded in participant’s interview booklet. While aided by the CIS-R interview questions to make a diagnosis, clinicians formed a diagnosis independently of CIS-R score. During the trial, clinicians were not provided the CIS-R diagnosis for their patients, which was based on its scoring algorithm. The CIS-R scoring took place during the data analysis.

The CIS-R (Lewis et al., 1992) was used to assess the presence of symptoms of common mental disorders. In the CIS-R, there are 14 different symptom groups which participants were asked to consider, regarding the last month prior to the interview, focusing on symptoms experienced within the last week. The 14 symptoms enquired after were: (1) Somatic symptoms; (2) Fatigue; (3) Sleep problems; (4) Irritability; (5) Physical health worries; (6) Depression; (7) Depressive ideas; (8) Worry; (9) Anxiety; (10) Phobias; (11) Panic; (12) Compulsive behaviours; (13) Obsessive thoughts; (14) Forgetfulness/concentration problems. Scores on each symptom group ranged from 0 to 4 (and 0 to 5 for depressive ideas), and Total CIS-R Scores are the sum of each symptom group with higher scores indicating higher levels of symptomatology.

The following were secondary outcomes:

1. Changes from baseline to the 6-month follow-up assessment in the disability of participants, as measured by WHO Disability Assessment Schedule 2.0 (WHODAS 2.0) [2].
2. Quality-adjusted life year at 6-month follow-up, as computed using the European Quality of Life Scale (EQ‐5D-3L) [3].

Attempts were made to collect reasons for treatment discontinuation and loss to follow-up.

### Translation and Back-translation

The GHQ-12, HoNOS, WHODAS 2.0, and EQ-5D in Bahasa Indonesia existed prior to the conception of this trial. The Bahasa Indonesia version of the GHQ-12, HoNOS, and WHODAS 2.0 were obtained from the National Institute of Health Research and Development, Ministry of Health, Indonesia. The Bahasa Indonesia version of the EQ-5D was obtained from the Euroqol Group. The CIS-R was translated into Bahasa Indonesia by Ms Wulansari Ardianingsih, MPhil (*Cantab*), MPsi (*UIndonesia*), and back-translated into English by the Corresponding Author.

### Researcher training

I distributed open recruitment flyers for research assistants, around Universitas Gadjah Mada campus and through social media groups. I invited 30% of applicants to an interview and recruited 28 research assistants (approximately 10% of all applicants) for the substantive study. Given the tremendous amount of interest, research assistants were selected based on a balance of high educational qualification with people skills and arithmetic ability, filtering in mainly recent medical graduates and social science master degree holders.

I recruited and trained 28 research assistants for the pilot study, and again for the substantive study, to distribute and score the screening questionnaires (GHQ-12) to all adult patients at our research sites. Research assistants were introduced to the background and aims of the research project, as well as detailed screening procedures. They were given a detailed flowchart of the procedure both hardcopy and digitally. They were also informed of the random audits which would be conducted during the period of the project.

For the substantive study, two research assistants were placed on duty per *Puskesmas*. Each dyad consists of a junior doctor and either a medical student or registered nurse. The data collection period coincided with the period where recent medical graduates in Yogyakarta and surrounding areas wait for news of their residency placements, leaving many free to take part as research assistants for our project.

### Sample size calculation

First, the sample size for non-inferiority individual randomisation RCT is calculated as a reference, using the estimated mean total HoNOS rating as a primary outcome [1]. The formula used is by Zhong, (2009). Assuming statistical significance value (α) of 0.05 and statistical power of 0.80, the standard deviation of 5.2, and clinical significance threshold (δ0) of 2 (Audin et al., 2001), the minimum sample size required for an RCT is 84. The standard deviation of 5.21 and clinical significance threshold used is the only available data on HoNOS reported from a sample of patients from eight National Health Service outpatient and community psychotherapy services in England. Total HoNOS scores range between 0 and 48, and a clinical significance threshold (δ_0_) of 2 could represent a plausible and realistic intervention effect. The minimum sample size using these estimates with a statistical bilateral significance value (α) of 0.05 and a statistical power of 0.80 is 84.

A larger sample size was needed to compensate for the clustering effect. For a trial with a fixed number of equal sized clusters (k), the required sample size per arm is n_c_, such that [21]:

n_c_ = $\frac{n_{i}k[1-\rho]}{[k-n_{i}\rho]}$

(1)

where *n*_i_ is the sample size required under individual randomisation and ρ is the intra-cluster correlation coefficient (ICC). The cluster randomisation might result in reduced efficiency and loss of power because the within-cluster responses tend to be more similar than those of individuals from different clusters (commonalities in the selection, exposure, shared environment, mutual interaction). A larger sample size was therefore needed to compensate for this clustering effect. Our approach is simplified because it does not consider variations in the number of participants in each cluster. Although this type of imbalance in cluster size may reduce the power of the trial, the loss is negligible for studies with more than 100 patients per arm [22]. Based on the additional assumption of an ICC of 0.1, the number of patients required would be 189 in each arm. Implementation research studies showed that in medical settings ICCs for outcome variables were generally lower than 0.05 [23]. In our trial, I decided to assume a high value for the ICC to consider a possible wide variation across different *Puskesmas*.

With an attrition rate of approximately 20%, I expected that a sample size of about 227 patients per arm (approximately 16 for each *Puskesmas*) should yield sufficient power.

Following the completion of baseline recruitment, a more accurate estimate of ICC can be calculated using the formula from Dr Yannan Jiang, University of Auckland. The formula [24] required a one-way analysis of variance, where the dependent variable is total HoNOS score, and the grouping variable is the cluster (*Puskesmas*).

$$ICC=\frac{(Between Groups Means Square-Within Group Means Square)}{(Between Groups Means Square+(number of participants per cluster-1)}$$

(2)

Given the new ICC derived from the trial dataset, the required sample size to keep a statistical power of 0.80 and a statistical bilateral significance value of 0.05 can be re-calculated.

## Analysis

### Intervention Uptake

Participant engagement with the treatment process was summarised and reported descriptively. There was no consensus regarding the appropriate number of therapy follow-ups (dose).

### Analysis of primary and clinical outcome measures

These analyses were carried out using the STATA software package, version 13. Cleaning of outcome and baseline data was conducted without the treatment group allocations in view. Summary statistics from these preliminary analyses were reviewed to identify data errors.

Preliminary analyses compared the characteristics of participants with and without complete data at six-months follow-up, by treatment group. They were carried out for the primary and secondary outcomes. This analysis was used to develop an understanding of the missing data mechanism and to determine the appropriate methods for dealing with missing outcome data.

Subsequent analyses took place with the valuable inputs and guidance of Senior Statistician at the University of Verona and Trial Manager: Dr Chiara Bonetto. The substantive study is a pragmatic cluster randomised controlled non-inferiority trial with the aim to assess the clinical effectiveness of two frameworks of primary mental health service provision in *Puskesmas* (community health centres) within a province, Yogyakarta. The unit of randomisation was the *Puskesmas* unit, and the unit of observation and analysis was the service users. The objective of conducting a non-inferiority trial was to demonstrate that neither the WHO mhGAP framework nor the Specialist framework was worse than the other with regards to clinical outcomes such as symptom severity and wellbeing.

Analyses were conducted using an intention-to-treat (ITT) approach. The effect of the type of service on HoNOS, EQ-5D and WHODAS 2.0 scores at six months were analysed separately in mixed-effects random regression models. Considering the trial design, in which patients (level 1) were nested within *Puskesmas* (level 2) [refer to the CONSORT guidelines for cluster randomised trials], the individual *Puskesmas* were included in the models as a random effect. Each model included treatment allocation and the baseline score of the outcome investigated as fixed effects.

In a secondary analysis, any missing data on follow-up outcomes were estimated using a multiple imputation approach by chained equations (MICE), which generated several different plausible imputed data sets and combined results from each of them. The predictive mean matching would be used to deal with possible non-normality when imputing continuous variables.

### Economic Evaluation

#### Perspective

The primary perspective of the economic evaluation was the Health Systems perspective, in line with the preference of NICE guidelines [25] and in line with the effort from national and provincial governments to provide universal health coverage to citizens. This study included only the use of mental health resources including *Puskesmas* follow-up services. While data on overnight hospitalisation and visits to outpatient general medical care were captured, they were not included in the analysis as they were not related to participants’ mental health.

At the follow-up, participants were asked to recall their use of health resources during the trial period (6 months). Differences in use of services between trial arms were compared and are reported for each service as the proportion of the group who had at least one contact. Statistical comparisons were not made to avoid problems of multiple testing and to keep the focus of the evaluation on costs and cost-effectiveness.

#### Costs

For each participant, a nationally applicable unit cost was applied to each item of service use reported during the trial (at the follow-up interview), to calculate the total cost for the duration of the trial. All costs are reported in Rupiah at 2017 prices. Discounting was not relevant as the follow-up did not exceed 12 months. Unit costs for primary care outpatient and inpatient services were obtained from a provincial cost calculation conducted in 2012 and adjusted for inflation based on national inflation rates https://www.statista.com/statistics/320156/inflation-rate-in-indonesia.

This trial used Yogyakarta health services unit cost valuation from Fidiyawati (2013) which followed the methodology recommended by the Directorate of Community Health Service Insurance, Ministry of Health 2003. This study of 2012 values (though published in 2013) indicated that the average unit cost in Yogyakarta outpatient medical service was Rp 13,961 and inpatient Rp 93,052 (£1 ~ Rp 19000 in March 2018). Based on these 2012 valuations [26], the 2016 and 2017 values could be calculated by taking into account published inflation rates as displayed in Table 4 [27].

In the absence of empirical data, the crude unit cost of Clinical Psychology consultation in *Puskesmas* could only be estimated. Psychologists’ monthly pay set by the provincial government (Rp 3,000,000 effective January 2017) was divided by the average number of psychology consultations per month
(231 appointments in 2017; annual average of 2772 appointments per psychologist), the unit cost for psychology consultation is estimated to be Rp 12, 987. The cost to health services of each psychology consultation takes into account the retribution for infrastructure, ancillary workforce, and medical record administration, which in Yogyakarta is determined at Rp 2100 [28]. The total cost of clinical psychology consultation in 2017 is Rp 15,087 per appointment (Table 4).

To receive free psychological care, participants required a GP referral. For participants in the Specialist Arm, the cost at baseline is a composite of the cost of GP consultation (which includes retribution) and the unit cost of psychology consultation (without retribution) which amounts to Rp 30,468.

Table 4. Unit costs of *Puskesmas* Mental Health Services, considering published inflation rates

| **YEAR** | **INFLATION RATE** | **WHO mhGAP Consultation COST in Rupiah** | **Clinical Psychology Consultation**  **COST in Rupiah** | |
| --- | --- | --- | --- | --- |
| 2012 | 3.98% | 13961 | |  |
| 2013 | 6.41% | 14516 | |  |
| 2014 | 6.40% | 15447 | |  |
| 2015 | 6.36% | 16435 | |  |
| 2016 (Baseline) | 3.53% | 17481 | | Without retribution: 12987 |
| 2017 (Follow-up) | 4.02% | 18098 | | 15097 |

#### Analyses

The primary economic evaluation explored cost-effectiveness in terms of HoNOS, the primary outcome for the trial. A secondary cost-utility analysis explored effectiveness in terms of Quality-Adjusted Life Years (QALYs).

EQ-5D-3L utility scores were used to calculate QALY improvements during the period of the trial (6 months) using the area under the curve approach [29] and this formula:

$$QALY Improvement = \frac{{QALY}_{T0} +{QALY}_{T1}}{2} \times\frac{1}{2} years$$

(3)

Incremental cost-effectiveness ratios (ICERS), i.e. the additional cost of one intervention compared with another divided by the additional effect, were calculated based on parameter estimates from random-effects linear regression models that represent costs and both outcomes and take into account the clustered structure of these data. While the ICER allows costs and outcomes to be considered together in a decision-making context, it is calculated from four sample mean values and is therefore subject to statistical uncertainty. The uncertainty of these estimates was explored first, by bootstrapping 1000 resamples to generate a new distribution of estimates and plotting these onto a cost-effectiveness plane for interpretation and then, by constructing cost-effectiveness acceptability curves (CEAC). The CEAC is a plot of the probability of the intervention being cost-effective (y-axis) for a range of willingness to pay thresholds per unit improvement in outcome (x-axis) [30]. Initially, for the cost-utility analysis the WHO recommendation of three times GDP per capita [31, 32] or a calculation based on estimates of opportunity costs [33] were considered to approximate an Indonesian willingness-to-pay threshold, but recent research on the Indonesian willingness-to-pay threshold for medical intervention enabled a more exact analysis to be conducted [34].

## Cultural Challenges and Bias

Prior to the trial, SGA applied for Leave to Work Away from the University of Cambridge, in light of the fieldwork and visiting fellowship to be undertaken in Indonesia. As part of the application, SGA completed a Risk Assessment document, which helped me highlight several potential issues at the fieldwork location.

While SGA is an Indonesian, she had not lived in the country for 16 years prior to the first fieldwork visit to Yogyakarta. During this first fieldwork, there was a terrorist attack in Jakarta, where several terrorists bombed and shot at places and people with foreign attributes, e.g. Starbucks. SGA established a regular reporting schedule with TVB and CB2 in Cambridge, to ensure that safety issues could be immediately dealt with, and if required, a return to Cambridge arranged. Having to travel accompanied, part of the Risk Assessment compromise, would also limit SGA’s independence to travel between each fieldwork site, given the distance and remote location of many sites (up to four hours from the centre of Yogyakarta).

SGA was aware of my negatively perceived identity as a double minority in Indonesia: both in ethnicity and religion. As a non-medical doctor, SGA was also at the lower end of the social power when compared to research stakeholders: Muslim, Javanese, older medical doctors working as civil servants. Despite her Cambridge affiliation, SGA did not enjoy the “White Privilege” granted to lighter-skinned researchers when working in less developed context, such as the privileges granted to Japanese Dr Nozomi Sakata when conducting research in Tanzania [35]. Conversely, as a woman ethnic Chinese Indonesian researcher who does not wear a hijab, SGA was vulnerable to discrimination at my fieldwork sites. Since SGA could not show respect by speaking High Javanese, SGA had to be especially mindful of her attire and body language when interacting with stakeholders. Greeting older and important stakeholders, SGA bowed and touched her forehead to the back of their right hand. Towards research assistants, SGA had to balance authority with Javanese politeness, aware of the secondary status of a woman in Javanese culture [36].

SGA was also aware of the rent-seeking culture of the fieldwork location, which could potentially present ethical challenges related to bribery requests, as she represented a foreign university. Prior to each data collection period, SGA would have to renew the provincial and district research licenses in person, as well as renew stakeholders’ support for the project. In her role as permission seeker, stakeholders who acted as gatekeepers could potentially present challenges. In light of these potential challenges, SGA developed local collaborations and formed a fieldwork advisory team, spearheaded by the previous Dean of Universitas Gadjah Mada Faculty of Medicine (LT) and the current Director of the Centre for Public Mental Health (DS). LT is a respected figure in the medical community in Yogyakarta, and on the other hand, all Puskesmas psychologists were required to submit biannual reports to DS. Their presence in the advisory team was deemed helpful should issues arise locally during fieldwork.

As a trained psychologist, there was also a potential that SGA had implicit bias, favouring the Specialist treatment arm over the WHO mhGAP treatment arm. Implicit bias generally tends to favour our own ingroup, and could influence the way SGA conducted training, negotiation with clinicians, her assessment of treatment outcomes, and/or recommendations at the end of the trial. SGA ensured that all trainings prior to the pilot and substantive trials were conducted together for both *Puskesmas* doctors and psychologists. To avoid clinicians feeling as though they were pitted against each other, representatives from the Provincial Health Authority opened the training sessions and provided a closing address. While SGA orchestrated the follow-up effort, research assistants who were Javanese collected patient outcomes data, introducing themselves in High Javanese as an expression of respect. The Trial Manager (CB1) was an independent party who conducted the randomisation of clusters, supervised the data analysis, and performed the ITT analysis.

References

1. Wing J, Curtis R, Beevor A. HoNOS: Health of the nation outcome scales. Report on Research and Development London: Royal College of Psychiatrists. 1996.

2. Sousa RM, Dewey ME, Acosta D, Jotheeswaran AT, Castro-Costa E, Ferri CP, et al. Measuring disability across cultures--the psychometric properties of the WHODAS II in older people from seven low- and middle-income countries. The 10/66 Dementia Research Group population-based survey. Int J Methods Psychiatr Res. 2010;19(1):1-17. doi: 10.1002/mpr.299. PubMed PMID: 20104493; PubMed Central PMCID: PMCPMC2896722.

3. Oppe M, Devlin NJ, SZENDE A. EQ-5D value sets: inventory, comparative review and user guide: Springer; 2007.

4. Hass L, deGruy F, Haas L. Primary care, psychology, and primary care psychology. Handbook of primary care psychology. 2004:5-19.

5. Haas LJ. Handbook of primary care psychology: Oxford University Press; 2004.

6. Derksen J. Primary care psychologists in the Netherlands: 30 years of experience. Professional Psychology: Research and Practice. 2009;40(5):493.

7. Elder MQ, Silvers SA. The integration of psychology into primary care: Personal perspectives and lessons learned. Psychological Services. 2009;6(1):68.

8. Setiyawati D, Blashki G, Wraith R, Colucci E, Minas H. Australian experts' perspectives on a curriculum for psychologists working in primary health care: implication for Indonesia. Health psychology and behavioral medicine. 2014;2(1):970-82. doi: 10.1080/21642850.2014.951937. PubMed PMID: 25750829; PubMed Central PMCID: PMC4346017.

9. Psikolog PUSKESMAS: Kebutuhan dan Tantangan bagi Profesi Psikologi Klinis Indonesia (Clinical Psychologist in Indonesian Primary Health Care: Opportunities and Challenges) [Internet]. Yogyakarta: Universitas Gadjah Mada; 2011 [cited 22 June 2013]. Available from: <http://mgb.ugm.ac.id/media/download/pidato-pengukuhan/Page-27.html>

10. Gupta SK. Intention-to-treat concept: a review. Perspectives in clinical research. 2011;2(3):109.

11. Hohmann AA, Shear MK. Community-based intervention research: Coping with the “noise” of real life in study design. American Journal of Psychiatry. 2002;159(2):201-7.

12. Goldberg D, Williams P. General health questionnaire (GHQ). Swindon, Wiltshire, UK: nferNelson. 2000.

13. Beecham J, Knapp M. Costing psychiatric interventions: Gaskell London; 2001.

14. Chan A-W, Tetzlaff JM, Altman DG, Laupacis A, Gøtzsche PC, Krleža-Jerić K, et al. SPIRIT 2013 statement: defining standard protocol items for clinical trials. Annals of internal medicine. 2013;158(3):200-7.

15. Meng L, Chen D, Yang Y, Zheng Y, Hui R. Depression increases the risk of hypertension incidence: a meta-analysis of prospective cohort studies. J Hypertens. 2012;30(5):842-51. doi: 10.1097/HJH.0b013e32835080b7. PubMed PMID: 22343537.

16. Ginty AT, Carroll D, Roseboom TJ, Phillips AC, de Rooij SR. Depression and anxiety are associated with a diagnosis of hypertension 5 years later in a cohort of late middle-aged men and women. J Hum Hypertens. 2013;27(3):187-90. doi: 10.1038/jhh.2012.18. PubMed PMID: 22592133.

17. Clarke DM, Currie KC. Depression, anxiety and their relationship with chronic diseases: a review of the epidemiology, risk and treatment evidence. Med J Aust. 2009;190(7 Suppl):S54-60. PubMed PMID: 19351294.

18. Lewis G, Pelosi A. Manual of the revised clinical interview schedule (CIS-R). Institute of Psychiatry, London. 1990.

19. Tongsiri S, Cairns J. Estimating population-based values for EQ-5D health states in Thailand. Value Health. 2011;14(8):1142-5. doi: 10.1016/j.jval.2011.06.005. PubMed PMID: 22152185.

20. Yusof FA, Goh A, Azmi S. Estimating an EQ-5D value set for Malaysia using time trade-off and visual analogue scale methods. Value Health. 2012;15(1 Suppl):S85-90. doi: 10.1016/j.jval.2011.11.024. PubMed PMID: 22265073.

21. Hemming K, Girling AJ, Sitch AJ, Marsh J, Lilford RJ. Sample size calculations for cluster randomised controlled trials with a fixed number of clusters. BMC Med Res Methodol. 2011;11:102. doi: 10.1186/1471-2288-11-102. PubMed PMID: 21718530; PubMed Central PMCID: PMC3149598.

22. Guittet L, Ravaud P, Giraudeau B. Planning a cluster randomized trial with unequal cluster sizes: practical issues involving continuous outcomes. BMC Med Res Methodol. 2006;6:17. doi: 10.1186/1471-2288-6-17. PubMed PMID: 16611355; PubMed Central PMCID: PMCPMC1513250.

23. Campbell MK, Fayers PM, Grimshaw JM. Determinants of the intracluster correlation coefficient in cluster randomized trials: the case of implementation research. Clin Trials. 2005;2(2):99-107. PubMed PMID: 16279131.

24. Jiang Y. Cluster Randomised Controlled Trials. Stats 773: Design and Analysis of Clinical Trials2012.

25. Drummond M. Clinical Guidelines: A NICE Way to Introduce Cost-Effectiveness Considerations? Value Health. 2016;19(5):525-30. PubMed PMID: Medline:27565268.

26. Fidiyawati T. USULAN ANGGARAN BERBASIS UNIT COST DI PUSKESMAS JETIS KOTA YOGYAKARTA PROVINSI DAERAH ISTIMEWA YOGYAKARTA: Universitas Gadjah Mada; 2013.

27. Statista. Indonesia: Inflation rate from 2012 to 2022 (compared to the previous year) 2018 [cited 2018 14 March 2018]. Available from: <https://www.statista.com/statistics/320156/inflation-rate-in-indonesia/>.

28. Widodo S. Pengukuran Efisiensi Pada Puskesmas di Daerah Istimewa Yogyakarta. Yogyakarta: Universitas Gadjah Mada; 2016.

29. Manca A, Hawkins N, Sculpher MJ. Estimating mean QALYs in trial‐based cost‐effectiveness analysis: the importance of controlling for baseline utility. Health economics. 2005;14(5):487-96.

30. Fenwick E, Claxton K, Sculpher M. Representing uncertainty: the role of cost-effectiveness acceptability curves. Health Econ. 2001;10(8):779-87. Epub 2001/12/18. PubMed PMID: 11747057.

31. Rachapelle S, Legood R, Alavi Y, Lindfield R, Sharma T, Kuper H, et al. The Cost-Utility of Telemedicine to Screen for Diabetic Retinopathy in India. Ophthalmology. 2013;120(3):566-73. doi: 10.1016/j.ophtha.2012.09.002. PubMed PMID: WOS:000315738200021.

32. Eichler HG, Kong SX, Gerth WC, Mavros P, Jonsson B. Use of cost-effectiveness analysis in health-care resource allocation decision-making: How are cost-effectiveness thresholds expected to emerge? Value in Health. 2004;7(5):518-28. doi: DOI 10.1111/j.1524-4733.2004.75003.x. PubMed PMID: WOS:000223779700003.

33. Woods B, Revill P, Sculpher M, Claxton K. Country-Level Cost-Effectiveness Thresholds: Initial Estimates and the Need for Further Research. Value Health. 2016;19(8):929-35. Epub 2016/12/19. doi: 10.1016/j.jval.2016.02.017. PubMed PMID: 27987642; PubMed Central PMCID: PMCPMC5193154.

34. Kristina SA, Endarti D, Andayani TM, Rokhman MR. RELIABILITY AND VALIDITY OF HYPOTHETICAL SCENARIOS FOR ESTIMATING WILLINGNESS TO PAY (WTP) PER QUALITY ADJUSTED LIFE YEAR (QALY) IN INDONESIA SETTING. 2017. 2017:5. Epub 2017-12-01. doi: 10.22159/ijpps.2017v9i12.22657.

35. Naveed A, Sakata N, Kefallinou A, Young S, Anand K. Understanding, embracing and reflecting upon the messiness of doctoral fieldwork. Compare: A Journal of Comparative and International Education. 2017;47(5):773-92.

36. Smith-Hefner NJ. Women and politeness: The Javanese example. Language in Society. 1988;17(4):535-54.
