# Supplementary material for: Can General Practitioners manage mental disorders in primary care? A partially randomised, pragmatic, cluster trial
Source: PLoS One. 2019 Nov 7;14(11):e0224724. doi: 10.1371/journal.pone.0224724 (PMC6837310; doi:10.1371/journal.pone.0224724)
Supplement: S2 File — (DOCX) [file pone.0224724.s004.docx]

**S2 File. Detail on the Malaysian Value Set for EQ-5D-3L**

The Malaysian value set combines both the time trade-off TTO and Visual Analogue Scale using linear additive regression [78]. The value set includes the N3 model from the original UK Measure and Valuation in Health study. The N3 model adds an interaction variable to capture the effect of any dimension with severe health state.

The Malaysian Value set for the EQ=5D-3L is:

Malaysian score = 1 – (0.067*N2) – (0.116*N3) – (0.084*m1) – (0.191*m2) – (0.097*sc1) – (0.16*sc2) – (0.053*ua1) – (0.122*ua2) – (0.054*pd1) – (0,127*pd2) – (0.081*ad1) – (0.086*ad2)

where **mo** is mobility, **sc** is self-care, **ua** is usual activities, **pd** is pain and discomfort, and **ad** is anxiety and depression. Variable **m1** is 1 if mobility is level 2, and 0 otherwise; **m2** is 1 if mobility is level 3, and 0 otherwise; **sc1** is 1 if self-care is level 2, 0 otherwise; **sc2** is 1 if self-care is level 3, 0 otherwise; **ua1** is 1 if usual activities is level 2, 0 otherwise; **ua2** is 1 if usual activities is level 3, 0 otherwise; **pd1** is 1 if pain and discomfort is 2, 0 otherwise; **pd2** is 1 if pain and discomfort is 3, 0 otherwise; **ad1** is 1 if anxiety and depression is 2, 0 otherwise; **ad2** is 1 if anxiety and depression is 3, 0 otherwise. Variable **N2** is 1 if any dimension is level 2 and 0 otherwise and; **N3** is 1 if any dimension is level 3 and 0 otherwise.
