# Supplementary material for: Can General Practitioners manage mental disorders in primary care? A partially randomised, pragmatic, cluster trial
Source: PLoS One. 2019 Nov 7;14(11):e0224724. doi: 10.1371/journal.pone.0224724 (PMC6837310; doi:10.1371/journal.pone.0224724)
Supplement: S3 File — (DOCX) [file pone.0224724.s005.docx]

**S3 File. Sample Size Calculation**

The formula used is by Zhong, (2009). The standard deviation of 5.2 and clinical significance threshold used is the only available data on HoNOS reported from a sample of patients from eight National Health Service outpatient and community psychotherapy services in England (Audin et al., 2001). Total HoNOS scores range between 0 and 48, and a clinical significance threshold (δ_0_) of 2 could represent a plausible and realistic intervention effect. The minimum sample size using these estimates with a statistical bilateral significance value (α) of 0.05 and a statistical power of 0.80 is 84.

For a trial with a fixed number of equal sized clusters (k), the required sample size per arm is n_c_, such that [57]:

n_c_ = $\frac{n_{i}k[1-\rho]}{[k-n_{i}\rho]}$

(1)

where *n*_i_ is the sample size required under individual randomisation and ρ is the intra-cluster correlation coefficient (ICC). The cluster randomisation might result in reduced efficiency and loss of power because the within-cluster responses tend to be more similar than those of individuals from different clusters (commonalities in the selection, exposure, shared environment, mutual interaction). A larger sample size was therefore needed to compensate for this clustering effect. Our approach is simplified because it does not consider variations in the number of participants in each cluster. Although this type of imbalance in cluster size may reduce the power of the trial, the loss is negligible for studies with more than 100 patients per arm [45]. Based on the additional assumption of an ICC of 0.1, the number of patients required would be 189 in each arm. Implementation research studies showed that in medical settings ICCs for outcome variables were generally lower than 0.05 [46]. In this trial, we decided to assume a high value for the ICC to consider a possible wide variation across different *Puskesmas*.

With an attrition rate of approximately 20%, we expected that a sample size of about 227 patients per treatment arm (approximately 16 for each *Puskesmas*) should yield sufficient power.

Following the completion of baseline recruitment, a more accurate estimate of ICC can be calculated using the formula from Dr Yannan Jiang, University of Auckland. The formula [56] required a one-way analysis of variance, where the dependent variable is total HoNOS score, and the grouping variable is the cluster (*Puskesmas*).

$$ICC=\frac{(Between Groups Means Square-Within Group Means Square)}{(Between Groups Means Square+(number of participants per cluster-1)}$$

(2)
