# Supplementary material for: Can General Practitioners manage mental disorders in primary care? A partially randomised, pragmatic, cluster trial
Source: PLoS One. 2019 Nov 7;14(11):e0224724. doi: 10.1371/journal.pone.0224724 (PMC6837310; doi:10.1371/journal.pone.0224724)
Supplement: S4 File — (DOCX) [file pone.0224724.s006.docx]

**S4 File. Unit costs of Puskesmas mental health services**

Fidiyawati’s study of 2012 values (though published in 2013) indicated that the average unit cost in Yogyakarta outpatient medical service was Rp 13,961 and inpatient Rp 93,052 (£1 ~ Rp 19000 in March 2018). Based on these 2012 valuations [47], the 2016 and 2017 estimates could be calculated by taking into account published inflation rates [48].

Table 1. Unit costs of *Puskesmas* Mental Health Services, considering published inflation rates

| **YEAR** | **INFLATION RATE** | **WHO mhGAP Consultation COST in Rupiah** | **Clinical Psychology Consultation**  **COST in Rupiah** | |
| --- | --- | --- | --- | --- |
| 2012 | 3.98% | 13961 | |  |
| 2013 | 6.41% | 14516 | |  |
| 2014 | 6.40% | 15447 | |  |
| 2015 | 6.36% | 16435 | |  |
| 2016 (Baseline) | 3.53% | 17481 | | Without retribution: 12987 |
| 2017 (Follow-up) | 4.02% | 18098 | | 15097 |

In the absence of empirical data, the crude unit cost of Clinical Psychology consultation in *Puskesmas* could only be estimated using average pay and total number of appointments in 2017. The total cost is a composite of the crude unit cost in 2017 and retribution for infrastructure, ancillary workforce, and medical administration [49]. The total cost of clinical psychology consultation in 2017 is Rp 15,087 per appointment .

To receive free psychological care, a GP referral is required. For participants in the Specialist Arm, the cost at baseline is a composite of the cost of GP consultation (which includes retribution) and the unit cost of psychology consultation (without retribution) which amounts to Rp 30,468.
