# Supplementary material for: Can General Practitioners manage mental disorders in primary care? A partially randomised, pragmatic, cluster trial
Source: PLoS One. 2019 Nov 7;14(11):e0224724. doi: 10.1371/journal.pone.0224724 (PMC6837310; doi:10.1371/journal.pone.0224724)

**S5 File. Details of cost-effectiveness analysis and cost-utility analysis.**

Fig 1 shows the scatterplot of bootstrapped mean differences in costs and HoNOS outcome scores. The majority of the scatter points indicate that the Specialist framework is more effective than WHO mhGAP framework (to the right of the y-axis) and lie in the northeast quadrant (78%) where the Specialist framework is more effective but more costly, and the southeast quadrant (9%) where the Specialist framework is more effective and less costly. The remaining scatter points show poorer outcomes for the Specialist framework compared to the WHO mhGAP framework and fall in the northwest (12%; less effective, more costly) and southwest (1%; less effective, less costly) quadrants.

Fig 1. Bootstrapped mean differences in costs HoNOS scores of WHO mhGAP arm compared to Specialist arm at 6-month follow-up


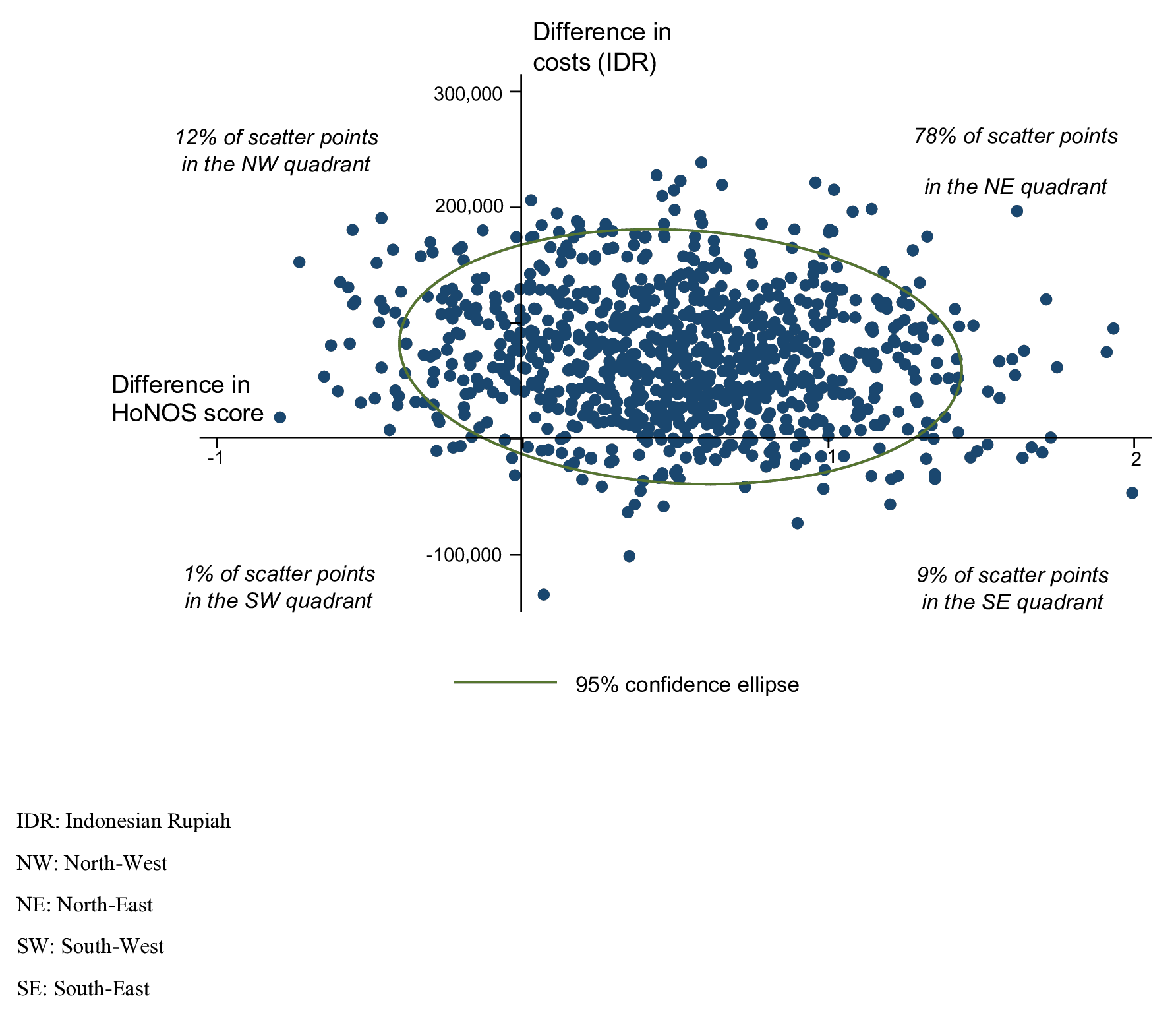


The CEAC for the primary analysis suggests that the probability of the Specialist framework being cost-effective compared to the WHO mhGAP framework ranges from just under 10% at a zero willingness to pay for a unit of improvement in HoNOS score to over 80% at a Rp 1,000,000 willingness to pay threshold (Fig 2).

Fig 2. Cost-effectiveness acceptability curve showing the probability that Specialist framework is cost-effective compared to WHO mhGAP for different values of willingness to pay thresholds using the HoNOS score at 6-month follow-up


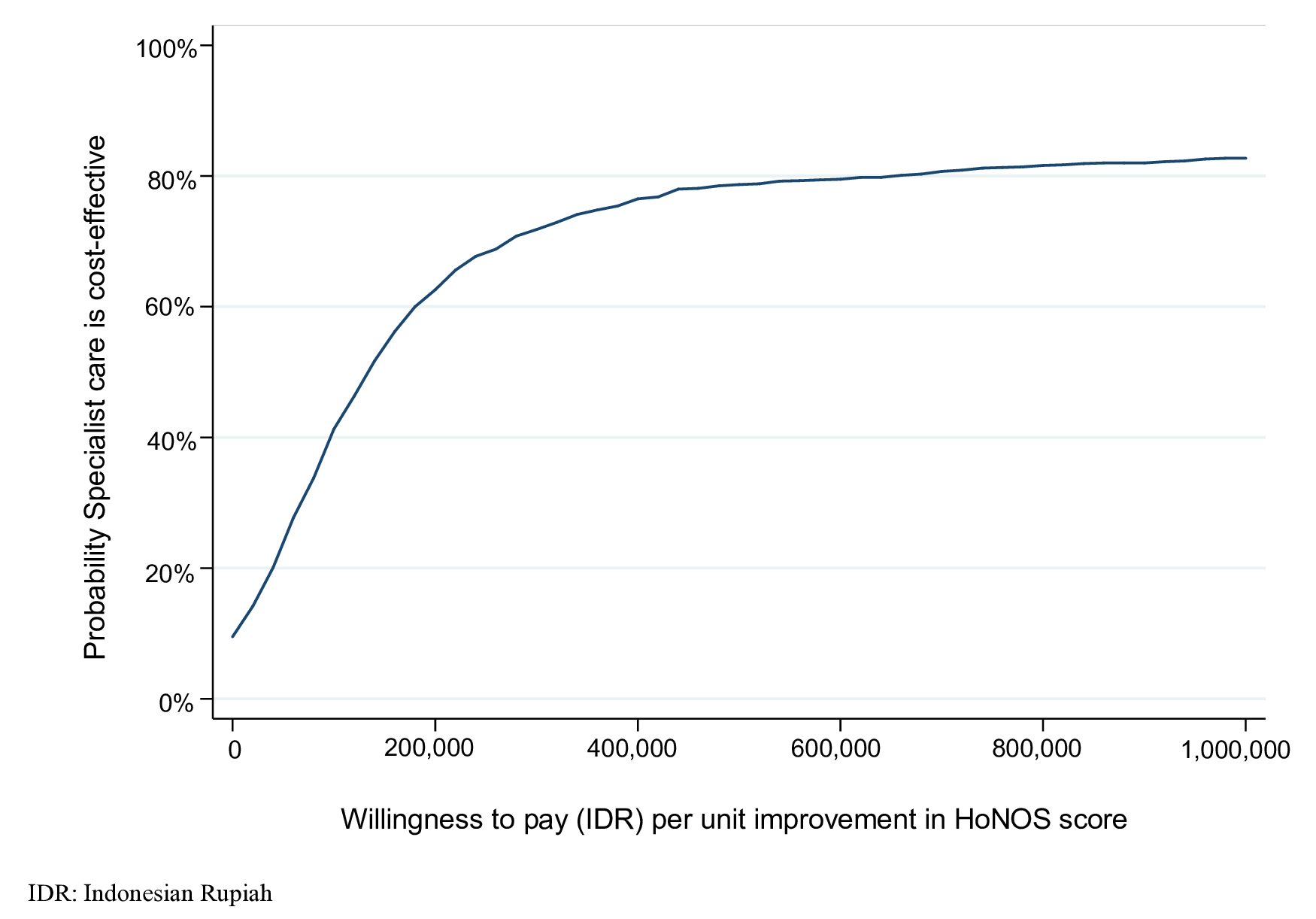


Fig 3 shows the scatterplot of bootstrapped mean differences in costs and QALYs. The majority of the scatter points indicate that Specialist framework is more effective than WHO mhGAP framework (to the right of the y-axis) and lie in the northeast quadrant (78%) where the Specialist framework is more effective but more costly, and the southeast quadrant (7%) where the Specialist framework is more effective and less costly. The remaining scatter points show poorer outcomes for Specialist framework compared to WHO mhGAP framework and fall in the northwest (14%; less effective, more costly) and southwest (2%; less effective, less costly) quadrants.

Fig 3. Bootstrapped mean differences in costs and QALYs of WHO mhGAP arm compared with Specialist arm at 6-month follow-up


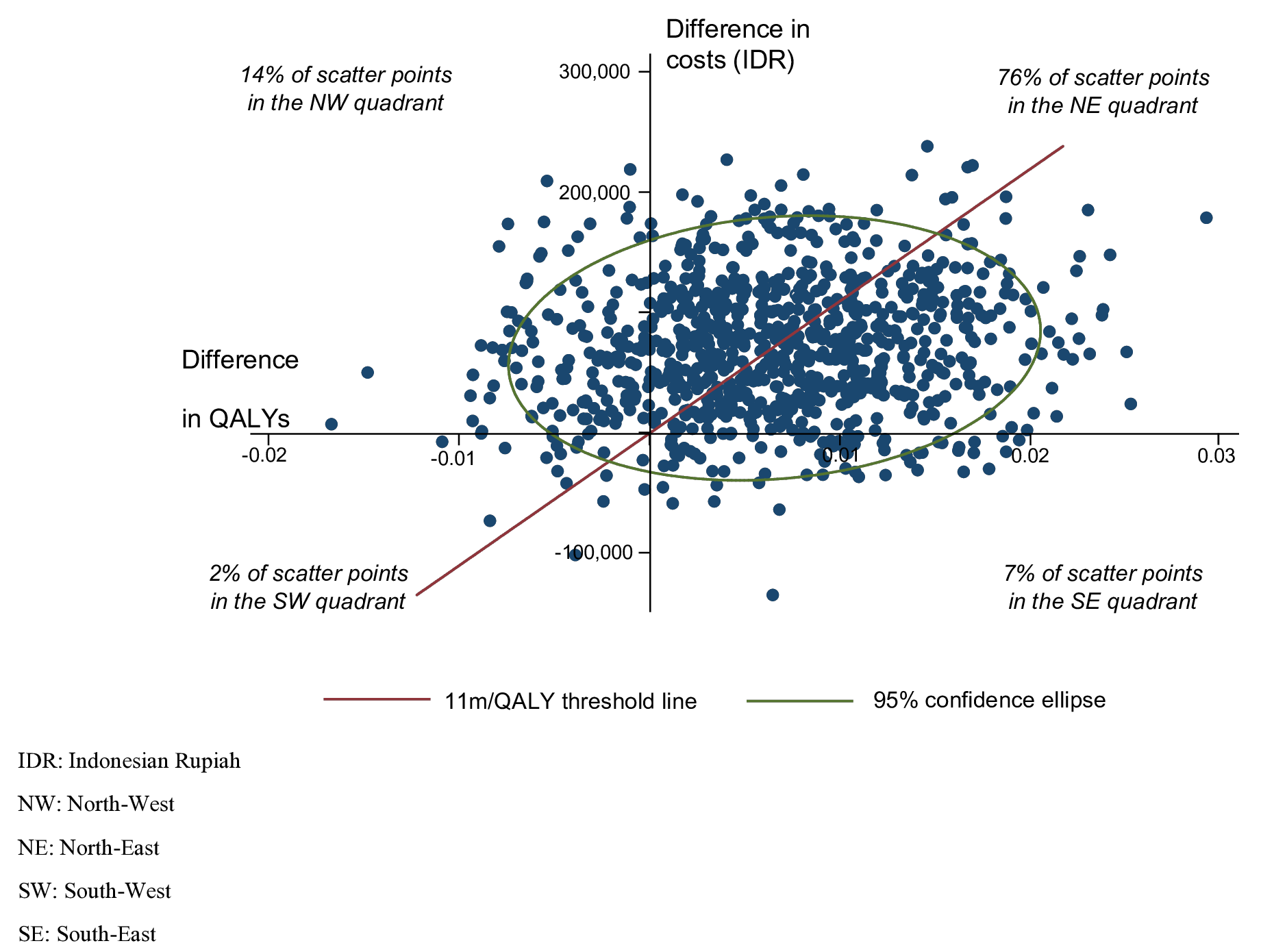


The cost-utility analysis (shown in Fig 4) suggest that Specialist framework has a 50% probability of being cost-effective compared to WHO mhGAP framework at the Indonesian willingness to pay for medical interventions of Rp 11,000,000 per QALY.

Fig 4. Cost-effectiveness acceptability curve showing the probability that Specialist framework is cost-effective compared to WHO mhGAP for different values of willingness to pay thresholds using QALYs at 6-month follow-up.


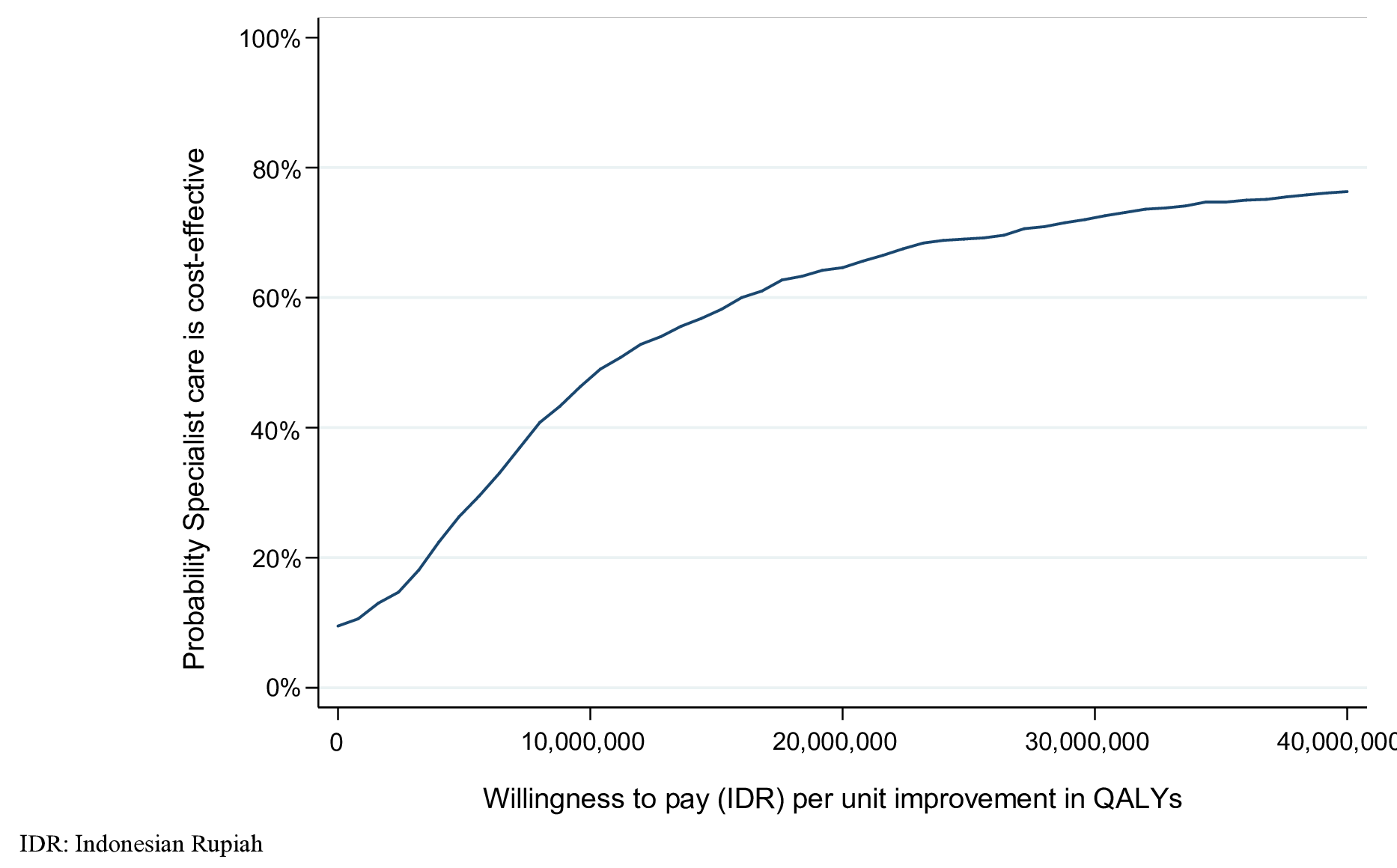

Supplement: S5 File — (DOCX) [file pone.0224724.s007.docx]
